# fIDBAC: A Platform for Fast Bacterial Genome Identification and Typing

**DOI:** 10.3389/fmicb.2021.723577

**Published:** 2021-10-18

**Authors:** Qian Liang, Chengzhi Liu, Rong Xu, Minghui Song, Zhihui Zhou, Hong Li, Weiyou Dai, Meicheng Yang, Yunsong Yu, Huan Chen

**Affiliations:** ^1^Department of Infectious Diseases, Sir Run Run Shaw Hospital, College of Medicine, Zhejiang University, Hangzhou, China; ^2^Hangzhou Digital-Micro Biotech Co., Ltd., Hangzhou, China; ^3^Ningbo Center for Disease Control and Prevention, Ningbo, China; ^4^Shanghai Institute for Food and Drug Control, NMPA Key Laboratory for Testing Technology of Pharmaceutical Microbiology, Shanghai, China; ^5^China National Accreditation Service for Conformity Assessment, Beijing, China; ^6^School of Medicine, Zhejiang University, Hangzhou, China; ^7^Zhejiang Chinese Medical University, Hangzhou, China

**Keywords:** bacterial genome, average nucleotide identity, k-mer, bacterial identification, strain typing

## Abstract

To study the contamination of microorganisms in the food industry, pharmaceutical industry, clinical diagnosis, or bacterial taxonomy, accurate identification of species is a key starting point of further investigation. The conventional method of identification by the 16S rDNA gene or other marker gene comparison is not accurate, because it uses a tiny part of the genomic information. The average nucleotide identity calculated between two whole bacterial genomes was proven to be consistent with DNA–DNA hybridization and adopted as the gold standard of bacterial species delineation. Furthermore, there are more bacterial genomes available in public databases recently. All of those contribute to a genome era of bacterial species identification. However, wrongly labeled and low-quality bacterial genome assemblies, especially from type strains, greatly affect accurate identification. In this study, we employed a multi-step strategy to create a type-strain genome database, by removing the wrongly labeled and low-quality genome assemblies. Based on the curated database, a fast bacterial genome identification platform (fIDBAC) was developed (http://fbac.dmicrobe.cn/). The fIDBAC is aimed to provide a single, coherent, and automated workflow for species identification, strain typing, and downstream analysis, such as CDS prediction, drug resistance genes, virulence gene annotation, and phylogenetic analysis.

## GRAPHICAL ABSTRACT

The framework of fIDBAC. fIDBAC platform contains a bacterial type-strain genome database, genome analysis pipeline, and separated analysis tools. Genome assemblies from the public database were quality checked and filtered. And the identification strategy was evaluated by three high-quality datasets—GEBA, FDA-ARGROS, and NCTC-3000 datasets.

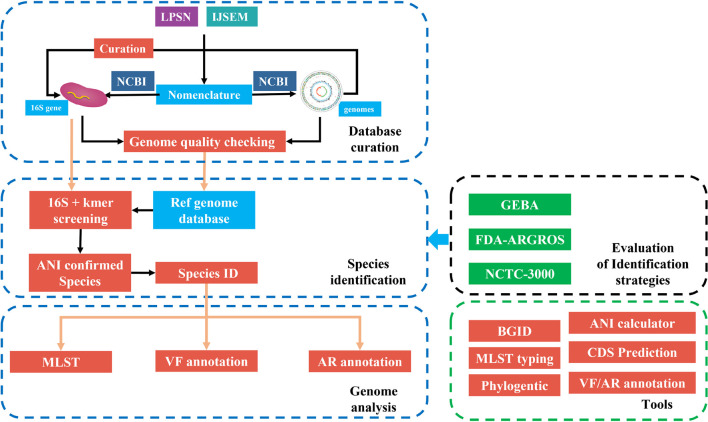

## Introduction

Accuracy in species identification is crucial for successful bacterial taxonomy, pathogen detection, and source tracking and is essential in the food industry, pharmaceutical industry, clinical diagnosis, and microbial resource development. Traditionally, bacterial identification relies on phenotypic identification, which suffers from being unable to be reproduced, labor-intensive, and time-consuming. Molecular methods tackle those drawbacks, and the 16S rRNA gene has becomes a popular molecular method in prokaryote taxonomy for its universal distribution in bacteria and archaea genomes and phylogenetic implication. Despite its large dataset, the 16S rRNA gene did not always possess enough resolution for species delineation, especially for closely related species (Botina and Sukhodolets, 2006; Chatellier et al., 2014). Besides, heterogenetic multicopy of the 16S rRNA gene in a genome hampers its application. Some other methods utilized additional

single-copy housekeeping genes to improve the accuracy of identification (Petti, 2007); however, the coverage of species is far too low compared with that of the 16S rRNA gene. Moreover, the latter might still be biased in gene selection and could not effectively work on all the taxa with a single workflow. It is expected that the more genome information used, the more accurate will be the taxonomy and identification. When a whole-genome sequence contains the full genetic information of a given taxon, it can effectively illustrate the species boundaries. Because of the benefits of next-generation sequencing, more bacterial genomes have been made available in public databases, which has recently led to a genome era in bacterial identification. However, identification tools, such as SpeciesFinder, Reads2Type (Larsen et al., 2014), TaxonomyFinder, and rMLST (Jolley et al., 2012), can only make use of reads or sequences that match 16S sequences or marker genes from genome sequencing data for identification. Expectedly, the average nucleotide identity (ANI) (Konstantinidis et al., 2006; Kim et al., 2014; Lee et al., 2016; Jain et al., 2018), based on the whole genome, can replace the present bacterial species delineation gold standard DNA:DNA hybridization (DDH) (Goris et al., 2007), which is robust even with draft genomes (Richter and Rosselló-Móra, 2009). ANI calculation suffers from computational resource and low speed due to a large amount of sequence alignment, and pairwise comparisons are not mentioned with thousands of genomes.

Another challenge for bacterial identification is the quality of the genome database. Firstly, genome sequences of type strain should be included in the database for class Qification and identification (Tindall et al., 2010). The type of a taxon, especially the type strain of species, which illustrates the full phenotypic and genotypic characteristics of original species description, plays an important role in phylogenetic analysis. Secondly, wrongly labeled genome sequences in a database pose a significant threat to identification. Errors might occur when a user submits a sequence from an isolate identified by a traditional procedure; also, contamination in a subculture or material transfer error occurs among different culture collections (Verslyppe et al., 2014). Thirdly, the completeness and contamination of genomes raise great attention (Parks et al., 2015) because they strongly bias the identification results. For example, contamination in genome sequences can lead to biased results with high ANI values between two distinct species. However, completeness and contamination of the draft genome might not be easily distinguished due to the high variation in genome size and gene content among species (Land et al., 2015). Lastly, the names might not match with the updated nomenclature from the International Code of Nomenclature of Prokaryotes (ICNP) (Parker et al., 2019).

In this study, we curated a bacterial type-strain genome database and developed a combined strategy for accurate and fast bacterial identification. Based on this, we developed a fast bacterial genome identification platform (fIDBAC), which integrates species identification, automated strain typing, and downstream analysis for bacterial genome sequences, in a coherent workflow. fIDBAC can be freely accessed at http://fbac.dmicrobe.cn/.

## Materials and Methods

The whole framework of the development of fIDBAC is illustrated in GraphicalAbstract.

### Genome Database Curation

Bacterial genomes with meta information such as “strain,” “culture collection,” “clone,” and “comments,” indicating the source strain of the genome, were collected from NCBI. The validly published bacterial names and type-strain list were taken mainly from LPSN (Parte, 2018), followed by checking with Bergey’s Manual of Systematic of Archaea and Bacteria (Whitman, 2016) and articles from *IJSEM*. Bacterial genomes of type strains were selected for database curation. Genomes labeled as synonyms were corrected according to the updated nomenclature.

Two methods were applied for genome sequence quality control. (i) CheckM (v1.0.18) (Parks et al., 2015), based on the lineage marker gene set, was used to evaluate the completeness and contamination of each genome. Assemblies with contamination of more than 5% or completeness of less than 90% were removed from the database. (ii) The 16S rRNA gene sequences extracted by RNAmmer (v1.2) (Lagesen et al., 2007) from genomes were aligned with the LTP database (version: LTPs132_SSU) (Yilmaz et al., 2014), to check the consistency. Assemblies with any disagreement at the genus level were excluded.

Finally, pairwise ANI calculation was performed between any two genomes to infer the wrongly labeled genomes. Different sequencing results (two or more assemblies) bearing one species name were clustered (by python package SciPy) based on ANI, and the distinguished outliers at the genus background were removed.

### Identification Strategy Development and Evaluation

For bacterial identification, we developed the following combined strategy:

1. 16S sequences extracted from queries were blasted against the LTP database.
2. K-mers extracted from the query genome were matched to the curated k-mer database by KmerFinder (v3.1).
3. The top 20 closest species were extracted from the results of the above-mentioned methods. Furthermore, the corresponding type-strain genomes were subjected to ANI calculation with the query genome by fastANI (v1.1).
4. Only the most closely related species (ANI > 95%) was reported.

Three datasets were used to evaluate the accuracy of fIDBAC identification strategy:

1. Genomic Encyclopedia of Bacteria and Archaea (GEBA) dataset. Nine hundred seventy-three bacteria assemblies from GEBA were selected for the test (30 archaeal genomes were removed from the test). GEBA releases 1,003 high-quality type-strain genomes including bacteria and archaea, which were sequenced by the Illumina HiSeq 2000 platform (Mukherjee et al., 2017).
2. FDA-ARGROS dataset. Five hundred twenty-three bacteria genomes without undefined labels (such as sp.) from the FDA-ARGROS project were downloaded on 2019.07.20 (Sichtig et al., 2019). FDA-ARGOS was aimed to provide well-quality-controlled reference genomes for diagnostic purposes, and the organism was confirmed by independent reference methods before sequencing. The FDA-ARGROS project mainly focuses on clinically relevant microorganisms and nearby neighboring species.
3. NCTC 3000 dataset. Nine hundred ninety-six genomes from the NCTC 3000 project^1^, with ambiguous records such as being labeled as sp., were removed. NCTC 3000 projects are working on producing complete genomes of the 3000 type and reference bacterial strains form PHE culture collection.

The best hits from the 16S rRNA alignment, KmerFinder with a complete genome database, KmerFinder with its type-strain genome database, and fIDBAC were compared in the above evaluation. Moreover, the respective effectiveness of screening the closest neighbors by 16S rRNA sequence similarity and KmerFinder shared k-mers was also evaluated in the fIDBAC identification strategy.

### Speed Test of Identification

The speed was tested on a tower server with an 86_64 architecture, 52 cores, 114 threads, and 256 GB RAM. Three hundred bacterial genome assemblies were randomly selected to test the identification speed.

### Web Interface Development

fIDBAC was established based on Python/Django and MySQL. All data in fIDBAC were stored and managed using MySQL (version 5.5.29). Additionally, the data processing programs were written in Python (version 2.7 and 3.6), and the web services were constructed using uWSGI + nginx.

Perl (v5.10) scripts were written to construct the bioinformatic pipeline. Multilocus sequence typing (MLST) was performed by BLAST (v2.4.0, e-value 1e–10) assembled genome sequences against the PubMLST database (Jolley et al., 2018) (with 133 species included) according to conventional seven-locus housekeeping gene schemes. Allele number and the sequence type (ST type) were assigned when the alignment coverage and identity reached 100%. Gene prediction was achieved by Prokka (v1.11) (Seemann, 2014). The predicted genes underwent BLAST analysis against the Comprehensive Antibiotic Resistance Database (CARD, v3.0.9) (Alcock et al., 2020) (111 drug classes included) and Virulence Factor Database (VFDB) (Liu et al., 2019) (909 virulence factors included), with the following parameters: e-value ≥ 1e–5, identity ≥ 80, and coverage ≥ 60. There are three ANI calculation methods incorporated in fIDBAC, namely, OrthANI (v1), gANI (Varghese et al., 2015), and fastANI. Single-nucleotide polymorphisms (SNPs) of assemblies were detected by Mummer (Kurtz et al., 2004) and were subjected to IQ-TREE (v1.6.6) (Minh et al., 2020) to automatically determine a prior substitution model for phylogenetic analysis. A maximum likelihood tree was constructed with a bootstrap of 100. Seventeen *Salmonella enterica* genomes mainly isolated from a food factory (Pightling et al., 2018), aimed to identify the resident pathogen, were taken from NCBI and used to run through the fIDBAC phylogenetic module.

## Results

### Overview of the Curated Database

**FIGURE 1:**
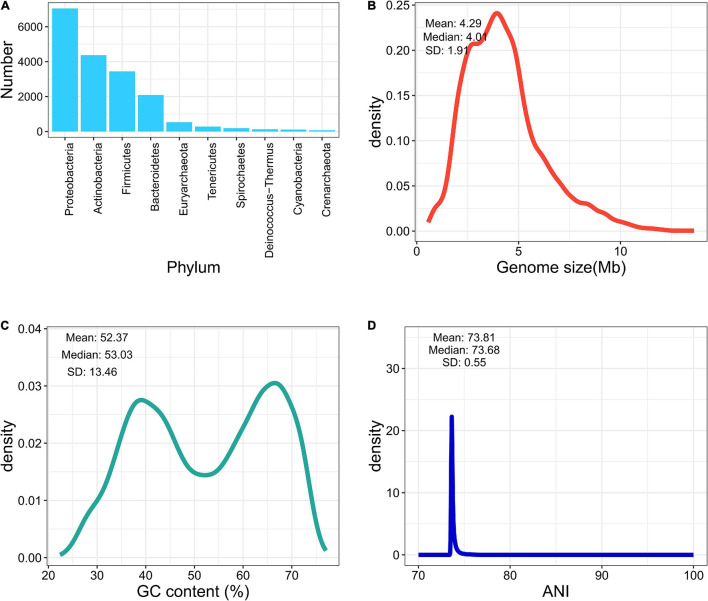
Brief summary of fIDBAC database. **(A)** Number of genomes in each phylum. **(B)** Distribution of genome size in the fIDBAC database. **(C)** Distribution of GC content in the fIDBAC database. **(D)** Distribution of pairwise ANI values between any of two assemblies in the fIDBAC database.

We collected 13,161 bacterial genome assemblies from NCBI, according to the type-strain list retrieved from LPSN and IJSEM. Three hundred and thirty-one genomes were removed (completeness of less than 90% or a contamination rate greater than 5%). Twelve genomes were excluded because of abnormal GC content or genome size. Furthermore, 15 genomes containing heterogenetic 16S sequences disagreed at the genus level and were discarded. While investigating the wrongly labeled genomes, among species containing more than one type of genomes, 20 genomes were found mistakenly labeled and removed, according to the dendrograms of pairwised ANI values and strain information from LPSN or IJSEM. Labels of 732 genomes were corrected according to the updated nomenclature. After the elimination of low-quality and mislabeled genomes, eventually, 12,783 genome assemblies are included in the final database, covering 9,827 species and 2,448 genera. The average completeness reached 99.14%, and the average contamination was less than 0.79%. Figures 1A–D summarize the landscape of the curated database. Most of the pairwise ANI values gathered at 70%–80%, indicating clear between-species boundaries in the curated database.

### Performance Evaluation of fIDBAC in Species Identification

Three datasets were used to evaluate the accuracy of the fIDBAC pipeline and database. Table 1 shows the test results of five methods with three datasets. The fIDBAC, considering a 95% threshold, achieved a total accuracy of 96.38%, slightly lower than the best-hit results reported by fIDBAC (97.35%) and outperformed the best-hit results from 16S rRNA sequence alignment by 30.93%, those from KmerFinder with complete genome database by 30.33%, and those from KmerFinder with type genome database by 15.48%. When compared, the performances of the 16S sequence alignment and fIDBAC pipeline were robust at the three datasets. However, the performances of the two KmerFinder methods are not consistent among the three datasets. For example, the accuracy of KmerFinder with complete genome was better at the FDA-ARGROS and NCTC 3000 datasets; in contrast, the KmerFinder with type genome database showed an inverse trend.

**TABLE 1:** Accuracy of different methods on GEBA, FDA-ARGROS, and NCTC 3000 datasets.

	Expected^a^	16S^b^	KmerFinder (Com)^c^	KmerFinder-(type)^d^	fIDBAC^e^	fIDBAC-ANI (95%)^f^
GEBA	973	617 (63.41%)	272 (27.95%)	908 (93.32%)	963 (98.97%)	963 (98.97%)
FDA-ARGROS	523	365 (69.79%)	490 (93.69%)	432 (82.06%)	501 (95.79%)	488 (93.31%)
NCTC-3000	996	649 (65.16%)	884 (88.76%)	676 (67.87%)	962 (96.58%)	951 (95.88%)
Total	2,492	1,631 (65.45%)	1,646 (66.05%)	2,016 (80.90%)	2,427 (97.39%)	2,402 (96.38%)

*^a^Expected, the number of queries genomes.*

*^b^16S, number of 16S top 1 result that matches the original label.*

*^c^KmerFinder (Com), number of KmerFinder top 1 result with its complete genome database that matches the original label.*

*^d^KmerFinder (type), number of KmerFinder top 1 result with its type strain genome database that matches 1he original label.*

*^e^fIDBAC, number of fIDBAC top 1 result with its curated genome database that matches the original label.*

*^f^fIDBAC, number of fIDBAC top 1 result with its curated genome database that matches the 95% ANI threshold and the original label.*

**FIGURE 2:**
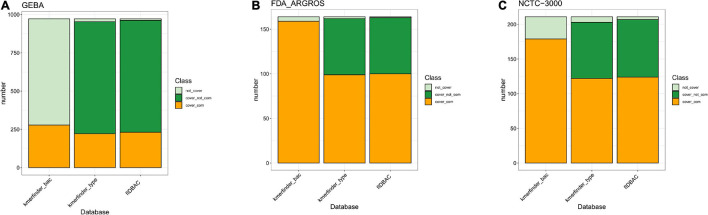
Bacterial species coverage in different methods compared to three datasets. **(A)** GEBA dataset; **(B)** FDA-ARGROS dataset; and **(C)** NCTC 3000 dataset. The color light green indicates the percentage of taxon from the corresponding dataset that was not covered by the reference database from each method. The percentage of taxon of the corresponding dataset covered by the reference database was divided into two parts, complete genomes (orange) and draft genomes (green). Kmerfinder_bac, the complete genome database from KmerFinder; kmerfinder_type, genomes of type strains from KmerFinder; fIDBAC, curated genomes of type strains from fIDBAC.

We further investigated the completeness of reference genome assemblies and taxonomy coverage in each database (Figures 2A–C). The type-strain database from KmerFinder covered 98.05, 98.78, and 96.21% of species in GEBA, FDA-ARGROS, and NCTC-3000 dataset, respectively, while the complete genome database had a lower species coverage with 28.6, 96.95, and 84.83%, respectively. However, if draft genomes are not taken into account, the situation becomes inverse. The complete genomes from a type-strain database of KmerFinder covered only 22.84, 60.37, and 57.82% of species in those three datasets. The differences indicated that only usage of the k-mer method could perform better with complete genomes as references.

**FIGURE 3:**
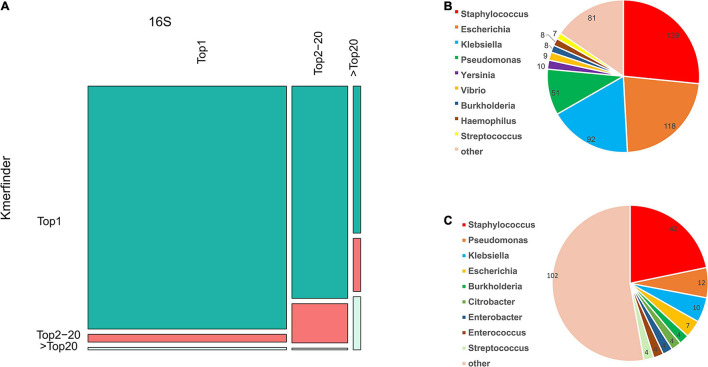
Comparison of accuracy in species identification by 16S and KmerFinder in GEBA, FDA-ARGROS, and NCTC-3000 datasets. **(A)** Mosaic plot of KmerFinder identification and 16S identification compared to the label in the fIDBAC database. **(B)** Species distribution of off-hit results of 16S identification. **(C)** Species distribution of off-hit results of KmerFinder identification.

Meanwhile, the species coverage of fIDBAC was quite similar to the type-strain database from KmerFinder, while fIDBAC identification was not significantly affected by draft genomes. fIDBAC identification employed additional screening steps. To evaluate the effectiveness of each screening step, we analyzed the top hits from the 16S rRNA sequence alignment and KmerFinder based on the fIDBAC database. Figure 3A summarizes the evaluation results. Most of the expected targets match the best hits reported by both 16S rRNA sequence alignment and KmerFinder. The 16S rRNA sequence alignment shows a limited complementary role for screening. ANI comparisons between the query genome and candidate genomes finally improve the accuracy. Collectively, the screening effectiveness of KmerFinder outperforms that of the methods based on 16S rRNA gene similarity. The genus distribution in wrong identification results was quite similar between the 16S method and KmerFinder methods (Figures 3B,C), indicating a further improvement in quality for those genomes.

**FIGURE 4:**
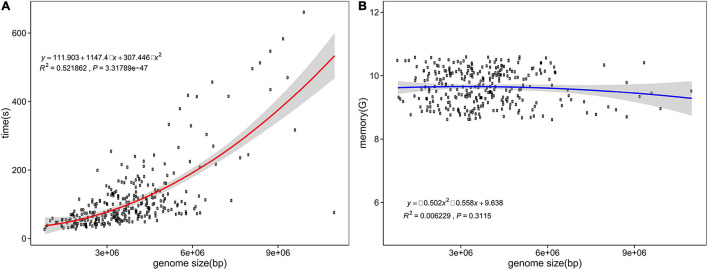
Time and memory consumption of fIDBAC identification. X axis, genome size (bp); y axis, consumed time **(A)** and memory usage **(B)**.

Computing resource consumption is shown in Figures 4A,B. According to the statistics of 300 randomly selected assemblies ranging from 0.82 to 10.99 Mb, the peak RAM usage reached 10.59 GB, with an average of 9.63 GB. The memory consumption of fIDBAC identification varied slightly when the genome size increases. The time spent was in the range from 0.45 to 11.01 min, with an average of 1.89 min, and was positively correlated with size of the query genome. For test assemblies < 5 Mb, the total time spent was less than 5 min.

### Web Interface and Example Output

To facilitate microbiologists to use the curated database for bacterial genome analysis, we developed a web interface—fIDBAC.

The home page of fIDBAC contains a brief introduction. The “About” page summarizes the information of the curated genome database, including the numbers of covered species and genus, assemblies, top 10 phylum, the distribution of ANI value by pairwise calculation between any two genomes, the genome size range, and GC content distribution, which were also displayed and updated regularly as required.

**FIGURE 5:**
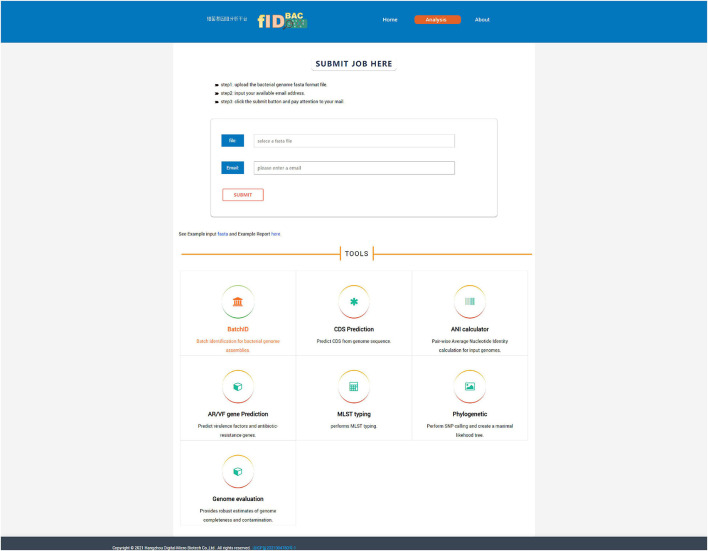
Screenshot of the “Analysis” page. One can submit a full pipeline analysis job here. Alternatively, the link for individual tools leads the user to the corresponding analysis.

The use of a web interface is mainly involved in the “Analysis” page (Figure 5). The “Analysis” page offers the most important function of fIDBAC. It integrates species identification, strain typing, and downstream analysis such as gene prediction, drug resistance gene annotation, and virulence gene annotation into a single, automatic workflow. “Analysis” accepts a pre-assembled bacterial genome as input. Species identification will be performed automatically after the query genome is uploaded. Once the species are specified (ANI > 95%), it will be checked if the corresponding MLST scheme from the PubMLST database exists. If the MLST scheme exists, the ST number will be determined through a script written by us. Gene prediction was performed by Prokka. Then fIDBAC will subject gene sequences to BLAST against the CARD and VFDB to infer the antibiotic-resistant gene and virulent factor information; regardless of whether the MLST scheme exists or not, the result link will be sent to the e-mail address provided by the user after the completion of the analysis.

**FIGURE 6:**
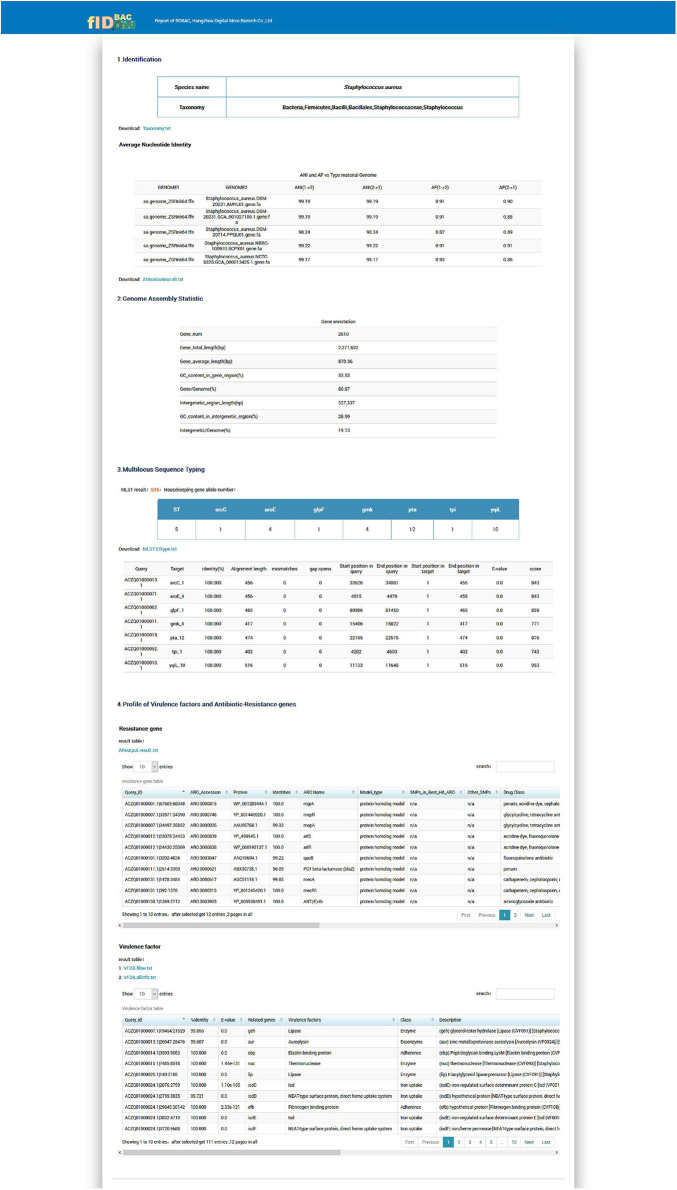
Screenshot of the example report by the full fIDBAC pipeline.

An example report for a *Staphylococcus aureus* genome is shown in Figure 6. The results report is composed of four parts.

The “Identification” illustrates the full taxonomy of the closest species (ANI > 95%).

The “Genome Assembly Statistic” includes the statistic of gene number, genome length, and GC% in gene region/intergenetic region.

The “Multilocus Sequence Typing” displays the sequence type and allelic profile when the identification matches a certain MLST scheme.

The “Profile of Virulence Factors and Antibiotic-Resistance Genes” provides a list of gene annotations of the query strain against the CARD and VFDB databases with thresholds described in the methods.

All the analysis results can be download in txt or png format.

The “Analysis” page contains seven tools, namely, “BatchID,” “CDS Prediction,” “ANI calculator,” “AF/VR Gene Prediction,” “MLST,” “Phylogenetic,” and “Genome evaluation.”

i. The “BatchID” tool supports batch genome file analysis and will return a simple list of identification results without the MLST and downstream analysis in the “Analysis” function.
ii. The “CDS Prediction” tool aims to predict genes from submitted genome assemblies.
iii. The “ANI calculator” tool calculates ANI among two or more uploaded genomes; the calculation methods include gANI (v1) and OrthANI, depending on the user’s choice. Results will include a matrix of pairwise ANI values, with a gradient color fill into each cell to map between any pair of genomes. This tool is especially helpful to investigate the relationship between specific isolates and type strains provide by the user.
iv. The “AF/VR Gene Prediction” tool aims to predict virulence factors and antibiotic-resistant genes in submitted genome assemblies.
v. The “MLST Typing” tool performs MLST typing as the user provides genome assembly and specifies a species.
vi. The “Phylogenetic” tool supports to perform pairwise SNP calling and generates a maximum likelihood tree with at most 30 genome sequences (Supplementary Figure 1). This tool is expected to play a role in strain typing and source tracking purposes.
vii. The “Genome evaluation” tool utilized CheckM to assess the completeness and contamination ratio for given assemblies.

## Discussion

A high-quality database of bacterial genomes and proper identification methods are essential for accurate identification. We curated a high-quality, less error-prone, and type-strain genome database by multi-step quality control. Though 16S rRNA alignment, ANI calculation and k-mer methods have been widely used in bacterial identification; however, they have several limitations. 16S rRNA alignment and k-mer methods are attractive because of their speed, but the best hits reported by them did not always match the target species. Even though the top 20 list was applied, there are still some cases that missed the target. On the other side, the best hit from 16S rRNA was generally closer to the real target, compared to KmerFinder (Supplementary Figure 2). These indicate that if identification by KmerFinder is not correct, it might lead to a more distant species assignment. Besides, k-mer methods lack a convenient threshold to circumscribe species boundaries as has been done by traditional DDH or ANI methods. Therefore, we combined all three methods to reduce the risks of missing the target. Test results show that the fIDBAC strategy achieves a high accuracy with time efficiency in bacterial identification.

The total of 90 inconsistent identifications in the test datasets was further investigated.

The insufficient coverage of bacterial species in the genome database contributed to the inconsistencies. Twelve inconsistent results were due to the lack of corresponding type-strain genome assemblies in the fIDBAC database. Presently, more than 16,000 names of bacteria have been validly published, while the number of available type-strain genomes is less than 10,000. Continuous efforts to fill the current gap are being made, such as GEBA and “The global catalog of microorganisms 10K type strain sequencing project (Wu and Ma, 2019).” Since 2018, IJSEM has required genome sequences of new taxa when published, and it is expected that the gap would not be enlarged. However, due to a lack of structured information for validated published names and sequences, curated bacteria genome databases must be reviewed periodically and manually updated to include newly available genomes. Low-quality genomes also hinder the species coverage for the database. Type-strain genomes for some of the species, such as *Ktedonobacter racemifer*, *Singulisphaera acidiphila*, *Terriglobus roseus*, *Sporomusa ovata*, *Promicromonospora kroppenstedtii*, *Deinococcus pimensis*, *Nevskia soli*, *Emticicia oligotrophica*, and *Duganella zoogloeoides*, are available in a public database but did not match the requirement of adequate completeness and low contamination rate and hence were discarded.

Moreover, the draft genomes, which might miss some key genetic information, affected the accuracy of identification. To save time, fast but less accurate methods are preferred for candidate screening. Recently, a published TYGS method (Meier-Kolthoff and Göker, 2019) screened closest neighbors by 16S rRNA comparison to avoid the time-consuming process of pairwise digital DDH with all genomes in the database. The risk of the sole usage of 16S rRNA is that the draft genomes might not contain available 16S rRNA sequences. For example, 345 (35.56%) draft assemblies in the GEBA dataset contain no 16S sequences. Additionally, the screening might involve a limited number of candidates, which might result in off-targets. K-mer methods like KmerFinder also have shortcomings. KmerFinder is biased to complete genomes. Apparently, complete genomes are less error-prone and should be a priority. However, draft genomes contribute 96.67% of all published genomes, and predictably, genomes of type or non-type strains would be still submitted mainly in the form of drafts. Benefiting from the combined screening strategy mentioned above, fIDBAC improves the ability to make use of drafts and complete genomes. It is expected that acquiring more complete genomes from type strains would additionally improve the accuracy of fIDBAC. An overview of the feature differences of the TYGS web server is provided in Table 2.

**TABLE 2:** An overview of the features of fIDBAC compared to the TYGS web server.

	TYGS	fIDBAC
Input	assembly (.fasta)	assembly (.fasta)
Primary search method	16S	k-mer and 16S
ORGI calculation method	dDDH	ANI
Species cutoff	70%	95%
Automatic MLST typing	NO	YES
AMR/VF gene prediction	NO	YES
Limits for user	publicly available	publicly available
Source code for local implement	NO	YES
Time consumed per assembly	about 60–80 min	about 5 min
Additional tools for genome evaluation and phylogenetic tree construction with custom genome	NO	YES

Almost half of the discordant results are related to wrongly labeled genomes from non-type strains. Forty genomes are not affiliated with labeled species, according to dendrograms based on pairwise ANI calculation (Supplementary Figures 3–27). Meanwhile, genomes from non-type strains are valuable for strain typing, source tracking, and other diverse study purposes. However, they suffer from low completeness, contamination, and mislabeling and need to be corrected or eliminated before application. The fIDBAC database, curated from type strains, could serve as a reference for the quality check of genomes from non-type strains. The curated procedure applied in fIDBAC might also be adequate to species without a type-strain genome. If wrongly labeled records are not taken into account in the total accuracy, both KmerFinder methods and fIDBAC method can improve by 2%–3% and fIDBAC methods can surpass 98% (Table 3).

**TABLE 3:** Revised accuracy of different methods on GEBA, FDA-ARGROS, and NCTC 3000 datasets.

	Expected^a^	16S^b^	KmerFinder (Com)^c^	KmerFinder (type)^d^	fIDBAC^e^	fIDBAC-ANI (95%)^f^
GEBA	973	617 (63.41%)	272 (27.95%)	908 (93.32%)	963 (98.97%)	963 (98.97%)
FDA-ARGROS	505	364 (72.08%)	478 (94.65%)	429 (84.95%)	501 (99.21%)	488 (96.63%)
NCTC-3000	967	644 (66.60%)	880 (91.00%)	671 (69.38%)	962 (99.48%)	951 (98.34%)
Total	2,445	1,625 (66.46%)	1,630 (66.67%)	2,008 (82.12%)	2,427 (99.26%)	2,402 (98.24%)

*^a^Expected, the number of query genomes.*

*^b^16S, number of 16S top 1 result that matches the original label.*

*^c^KmerFinder (Com), number of KmerFinder top 1 result with its complete genome database that matches the original label.*

*^d^KmerFinder (type), number of KmerFinder top 1 result with its type-strain genome database that matches the original label.*

*^e^fIDBAC, number of fIDBAC top 1 result with its curated genome database that matches the original label.*

*^f^fIDBAC, number of fIDBAC top 1 result with its curated genome database that matches the 95% ANI threshold and the original label.*

One of three inconsistencies indicated a requirement of taxonomy revisions for some species. Five of 90 inconsistent results were due to the existence of very closely related species (ANI > 95%), such as *Bordetella bronchiseptica* and *Bordetella parapertussis*, *Klebsiella oxytoca* and *Klebsiella grimontii*, *Chryseobacterium shigense* and *Chryseobacterium carnipullorum*, and *Bacillus megaterium* and *Bacillus aryabhattai*. Twenty-four of the assemblies were consistent at the best hit but did not match the threshold of 95%, which probably indicates a new taxon. The increasing bacterial genomes might help to find more previously defined species which are closely related and help us gain insight into the diversity of bacterial species. We believe taxonomic ambiguities will be finally resolved by microbiologists.

In this study, we present a free available web platform fIDBAC. fIDBAC provides a single, coherent, and automated workflow for fast species identification, strain typing, and downstream analysis including gene prediction, drug resistance gene/virulence factor annotation, and phylogenetic analysis. Future work will be focused on handling a larger database of soaring genome sequences and integrating more modules for bacterial genome analysis.

## Data Availability Statement

The code and accession numbers presented in the study are deposited in the Github repository (https://github.com/DMicrobe/fIDBAC).

## Author Contributions

HC, YY, and MY designed the study. QL, CL, and RX contributed to pipeline design, software installation and distribution, and data analysis and wrote and prepared the manuscript. MS, ZZ, HL, and WD contributed to data preparation and data analysis. All authors contributed to the article and approved the submitted version.

## Conflict of Interest

QL, CL, and HC were employed by the company Hangzhou Digital-Micro Biotech Co., Ltd. The remaining authors declare that the research was conducted in the absence of any commercial or financial relationships that could be construed as a potential conflict of interest.

## Publisher’s Note

All claims expressed in this article are solely those of the authors and do not necessarily represent those of their affiliated organizations, or those of the publisher, the editors and the reviewers. Any product that may be evaluated in this article, or claim that may be made by its manufacturer, is not guaranteed or endorsed by the publisher.

## Footnotes

1
https://www.phe-culturecollections.org.uk/products/bacteria/nctc-3000-project-a-comprehensive-resource-of-bacterial-type-and-reference-genomes.aspx

## Supplementary Material

Supplementary Figure 1 Example phylogenetic analysis of 17 *Salmonella* genomes from a factory.

Supplementary Figure 2 Distribution of ANI value between target and non-target top 1 results. Red, with 16S non-target top 1 results; Green, with KmerFinder non-target top 1 results. ANI calculation between the query assembly and type-strain assembly from the top 1 result of 16S alignment or KmerFinder was performed by fastANI.

Supplementary Figures 3–27 Dendrograms based on pairwise ANI values showed wrongly labeled genomes from the FDA-ARGROS and NCTC 3000 project. Genomes from FDA-ARGROS were colored green and marked by a superscript “F.” The superscript “F” was colored red if the genome was regarded as wrongly labeled. Genomes from the NCTC 3000 project were colored green and marked by a superscript “N.” The superscript “N” was colored red if the genome was regarded as wrongly labeled. Genomes without any superscript were derived from the fIDBAC curated database. Pairwise ANI calculation was performed by fastANI.
