# Supplementary figures and images for: fIDBAC: A Platform for Fast Bacterial Genome Identification and Typing
Source: Front Microbiol. 2021 Oct 18;12:723577. doi: 10.3389/fmicb.2021.723577 (PMC8558511; doi:10.3389/fmicb.2021.723577)

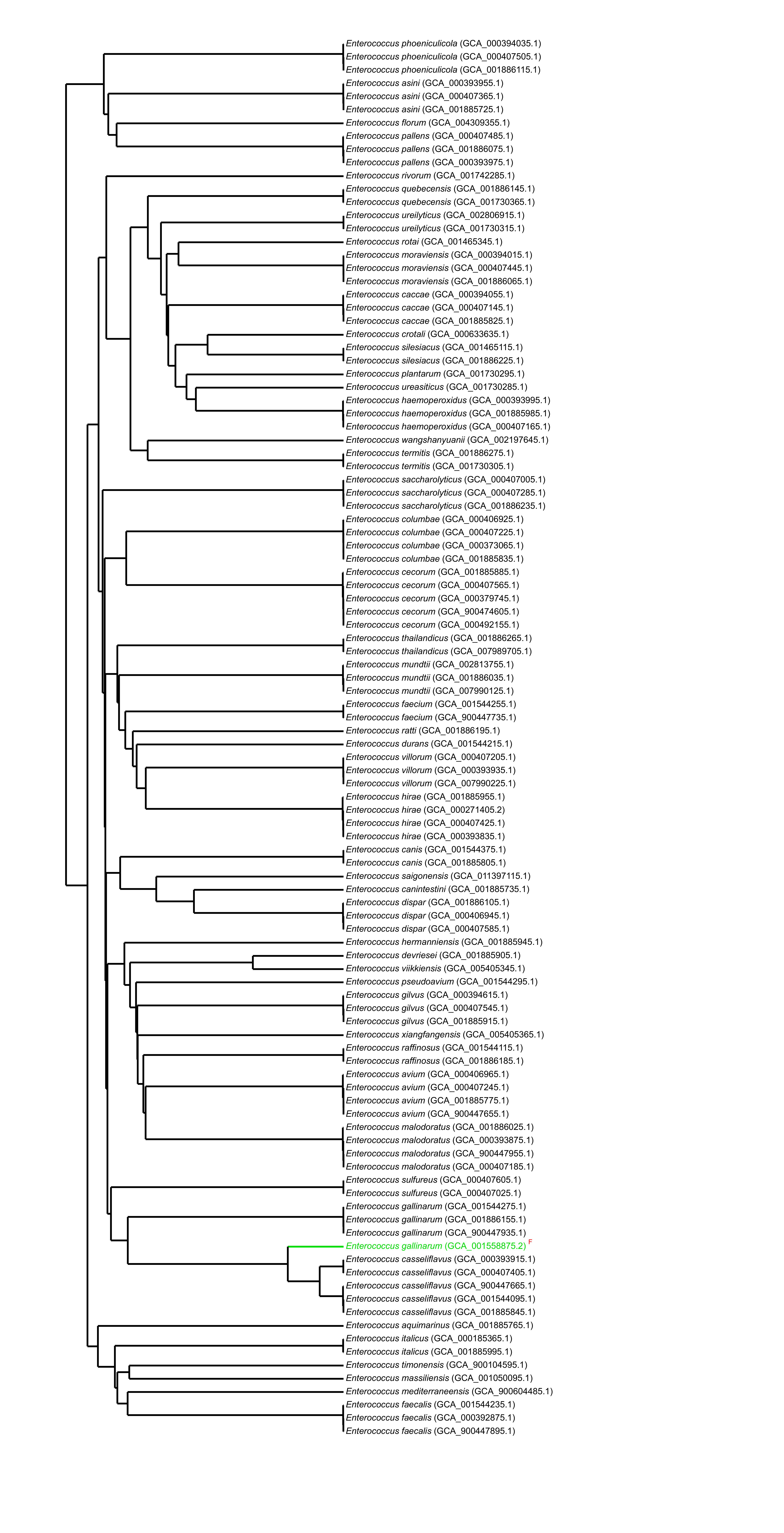

Supplement: Supplementary Figure 1 — Example phylogenetic analysis of 17 Salmonella genomes from a factory. [file Data_Sheet_1.zip › Supplementary Figure 8.Enterococcus.tiff]

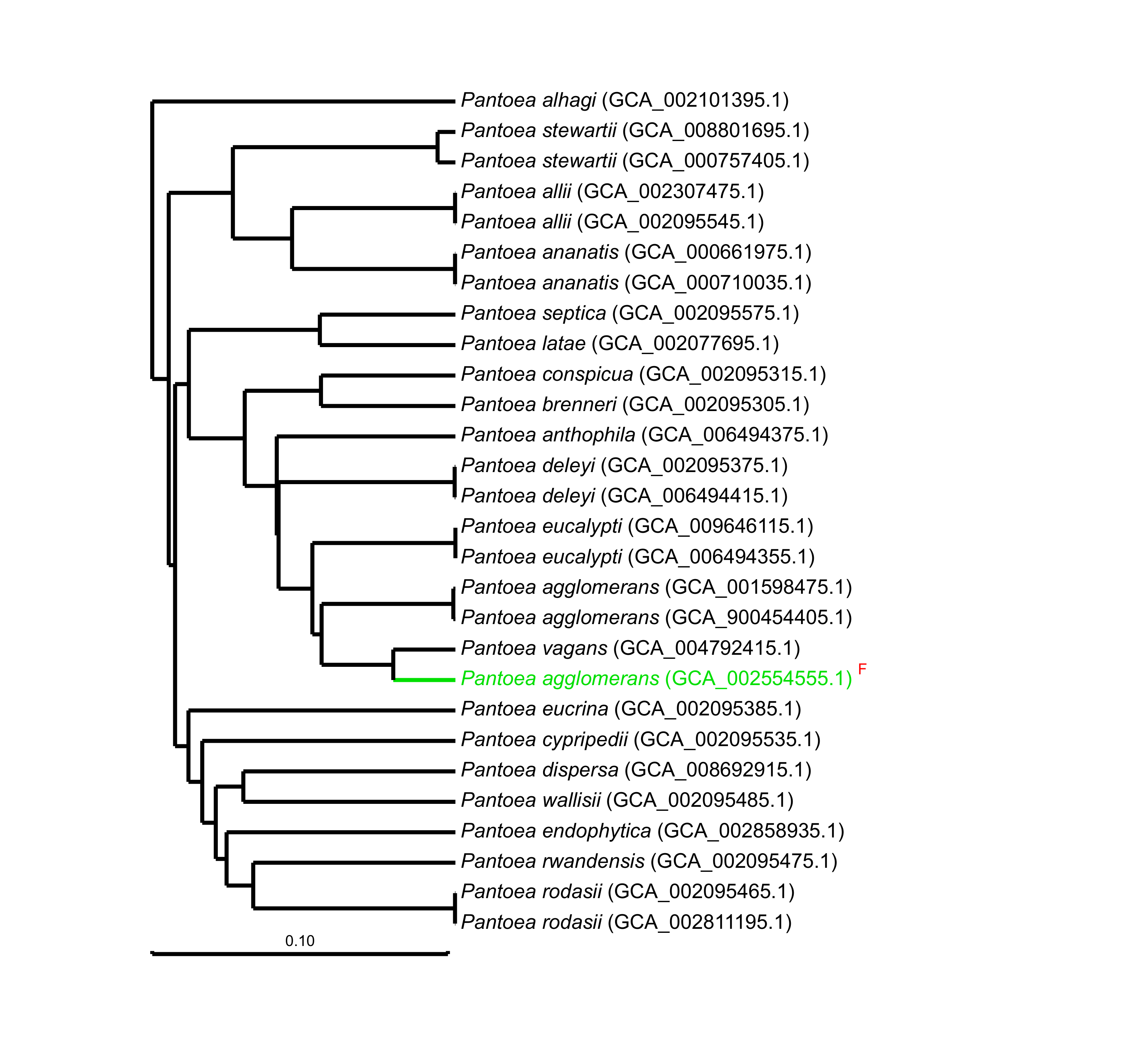

Supplement: Supplementary Figure 1 — Example phylogenetic analysis of 17 Salmonella genomes from a factory. [file Data_Sheet_1.zip › Supplementary Figure 9.Pantoea.tiff]

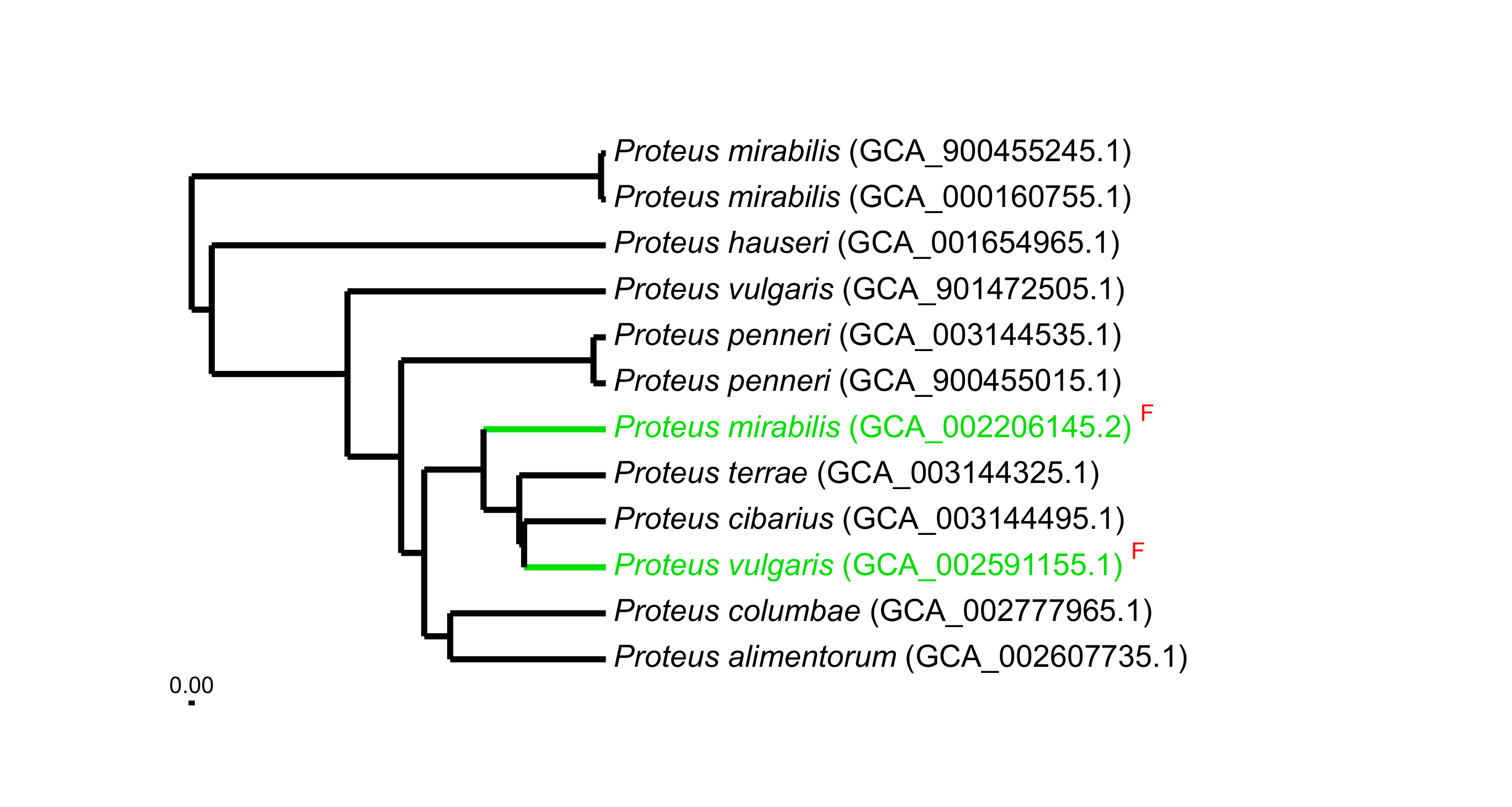

Supplement: Supplementary Figure 1 — Example phylogenetic analysis of 17 Salmonella genomes from a factory. [file Data_Sheet_1.zip › Supplementary Figure 10.Proteus.tiff]

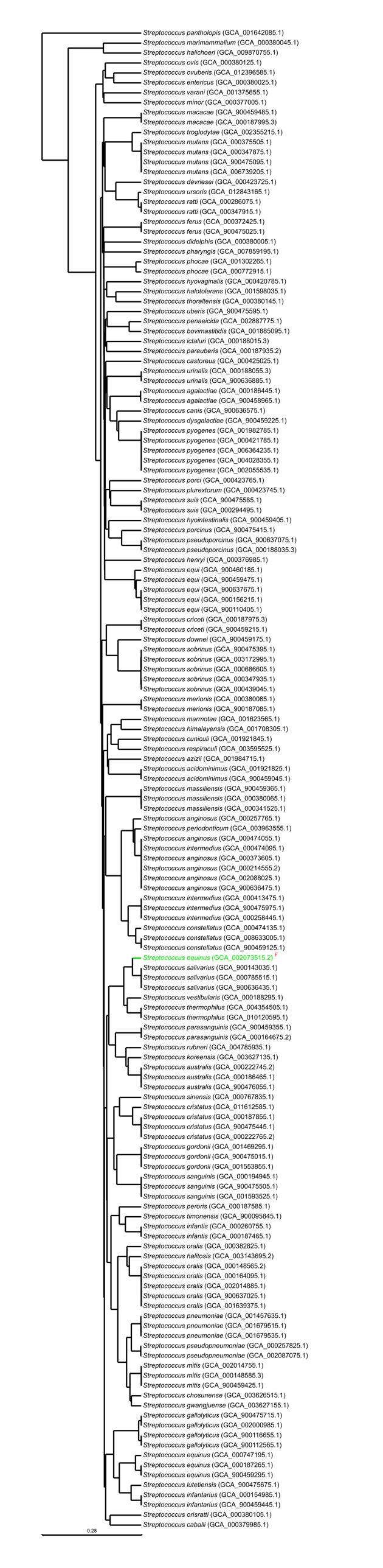

Supplement: Supplementary Figure 1 — Example phylogenetic analysis of 17 Salmonella genomes from a factory. [file Data_Sheet_1.zip › Supplementary Figure 12.Streptococcus.tiff]

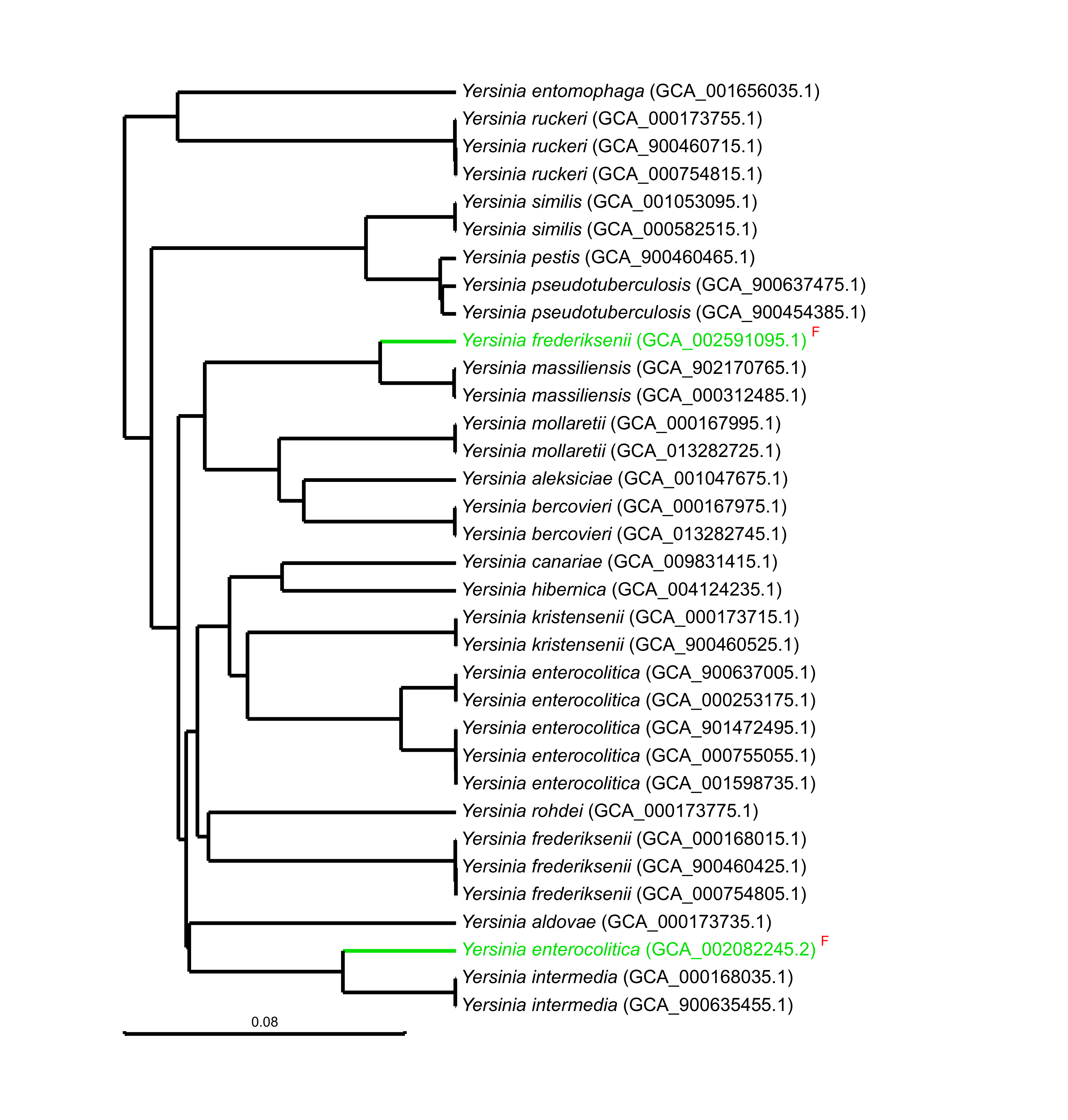

Supplement: Supplementary Figure 1 — Example phylogenetic analysis of 17 Salmonella genomes from a factory. [file Data_Sheet_1.zip › Supplementary Figure 13.Yersinia.tiff]

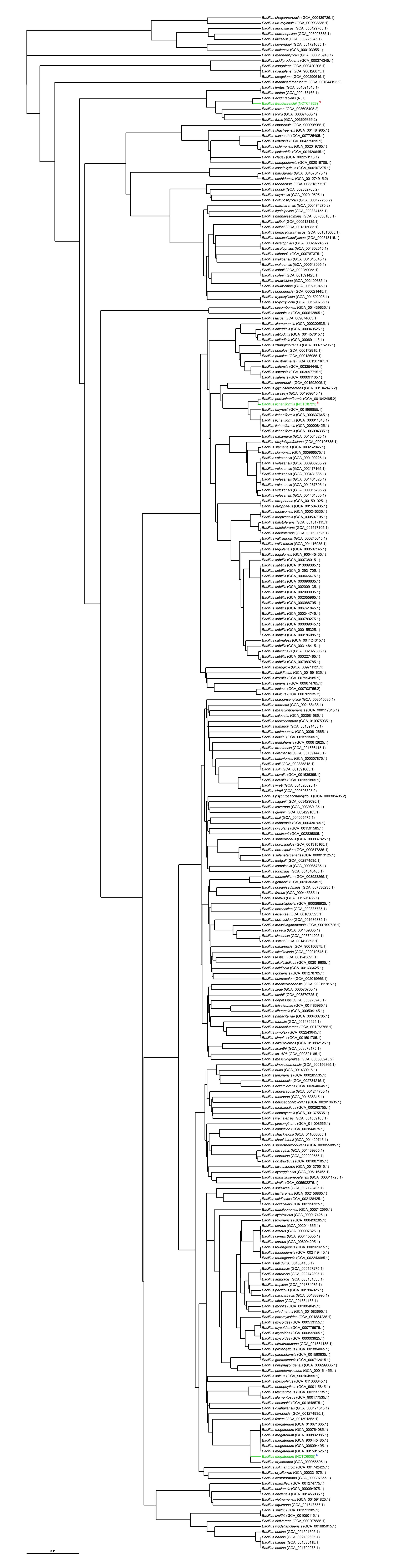

Supplement: Supplementary Figure 1 — Example phylogenetic analysis of 17 Salmonella genomes from a factory. [file Data_Sheet_1.zip › Supplementary Figure 14.Bacillus.jpg]

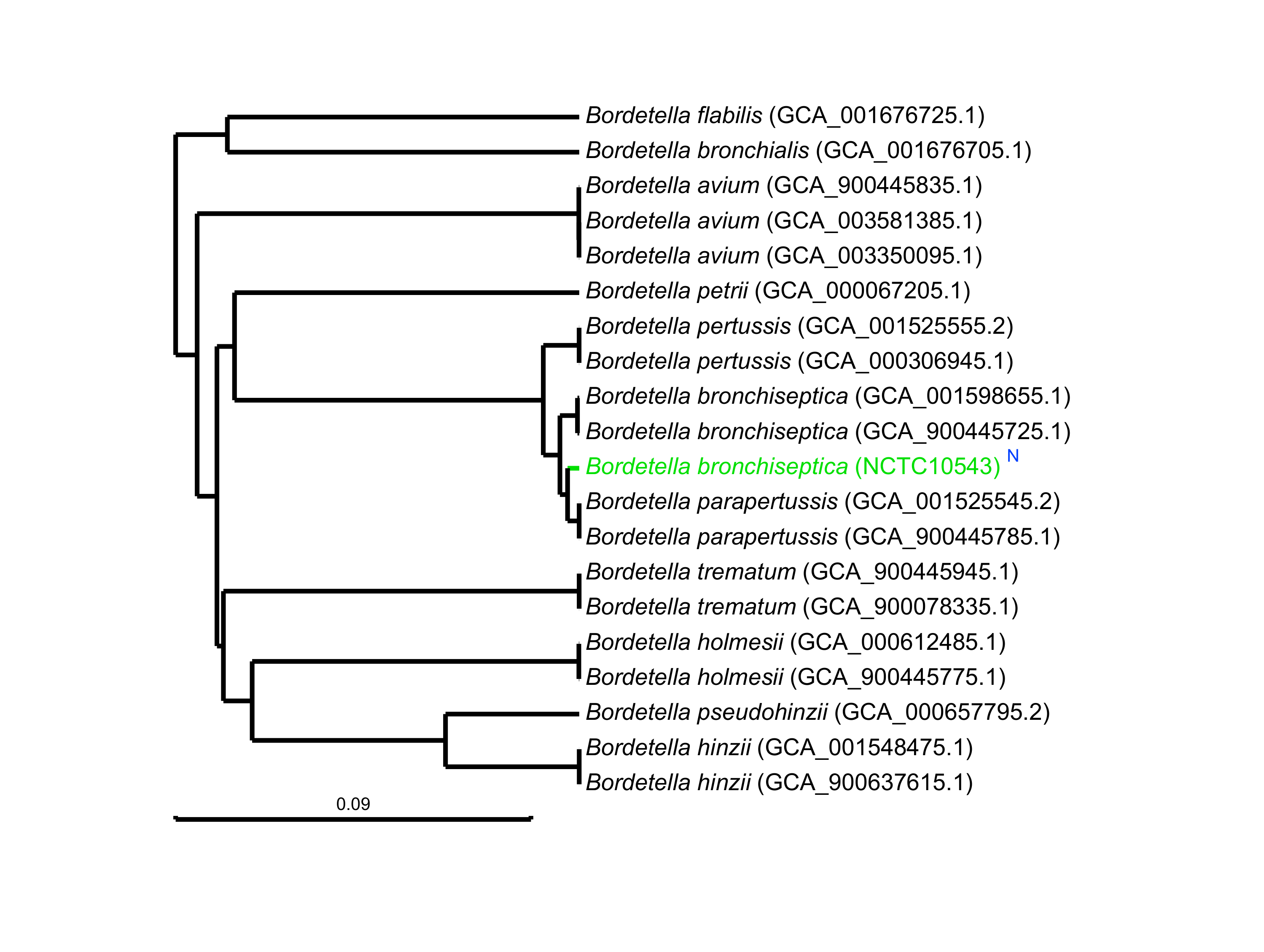

Supplement: Supplementary Figure 1 — Example phylogenetic analysis of 17 Salmonella genomes from a factory. [file Data_Sheet_1.zip › Supplementary Figure 15.Bordetella.tiff]

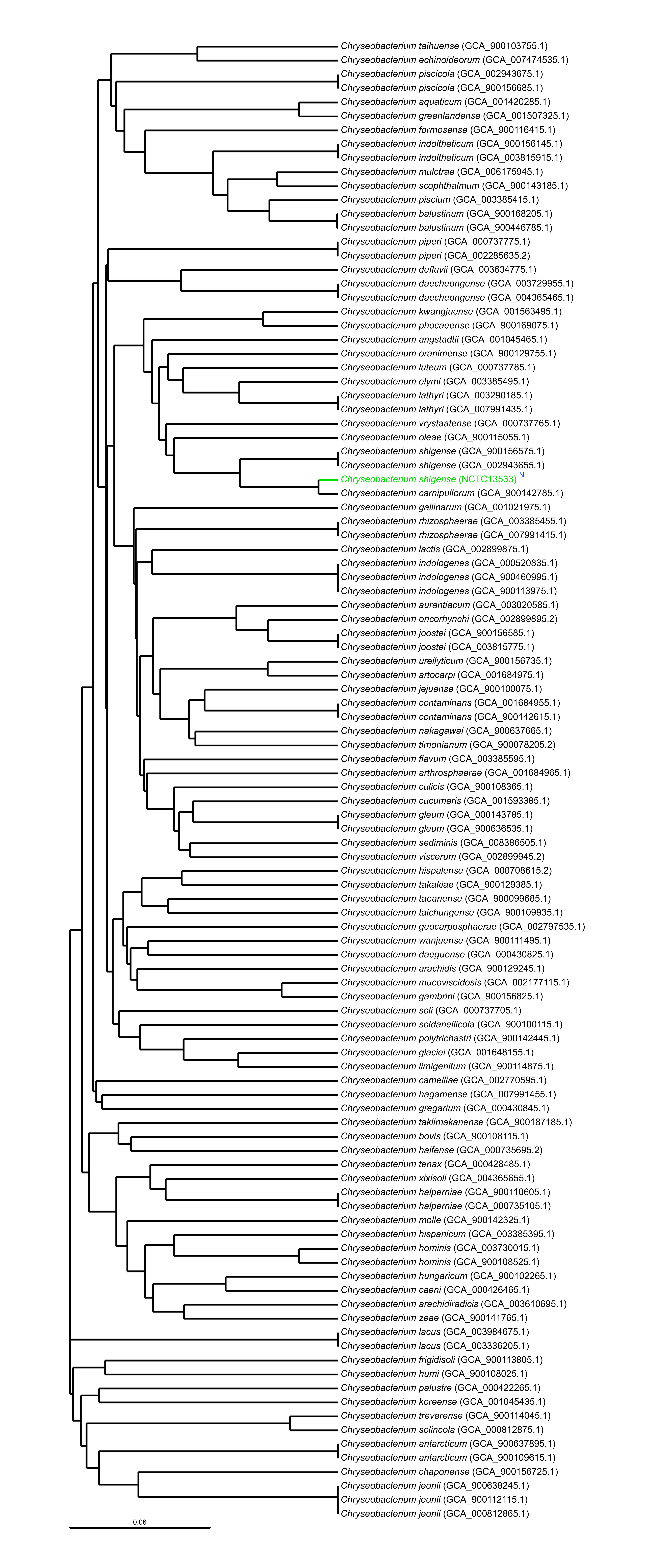

Supplement: Supplementary Figure 1 — Example phylogenetic analysis of 17 Salmonella genomes from a factory. [file Data_Sheet_1.zip › Supplementary Figure 16.Chryseobacterium.tiff]

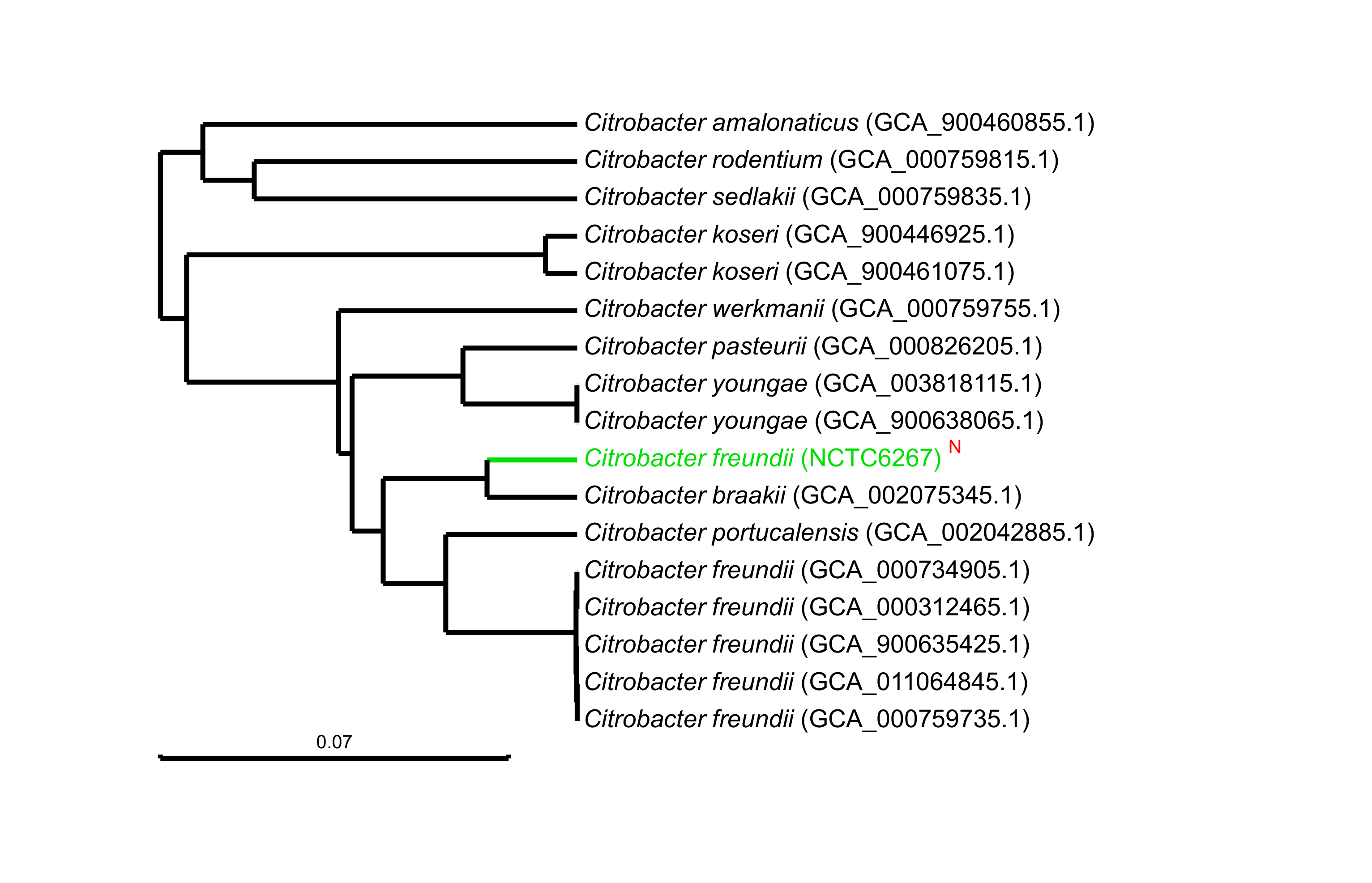

Supplement: Supplementary Figure 1 — Example phylogenetic analysis of 17 Salmonella genomes from a factory. [file Data_Sheet_1.zip › Supplementary Figure 17.Citrobacter.tiff]

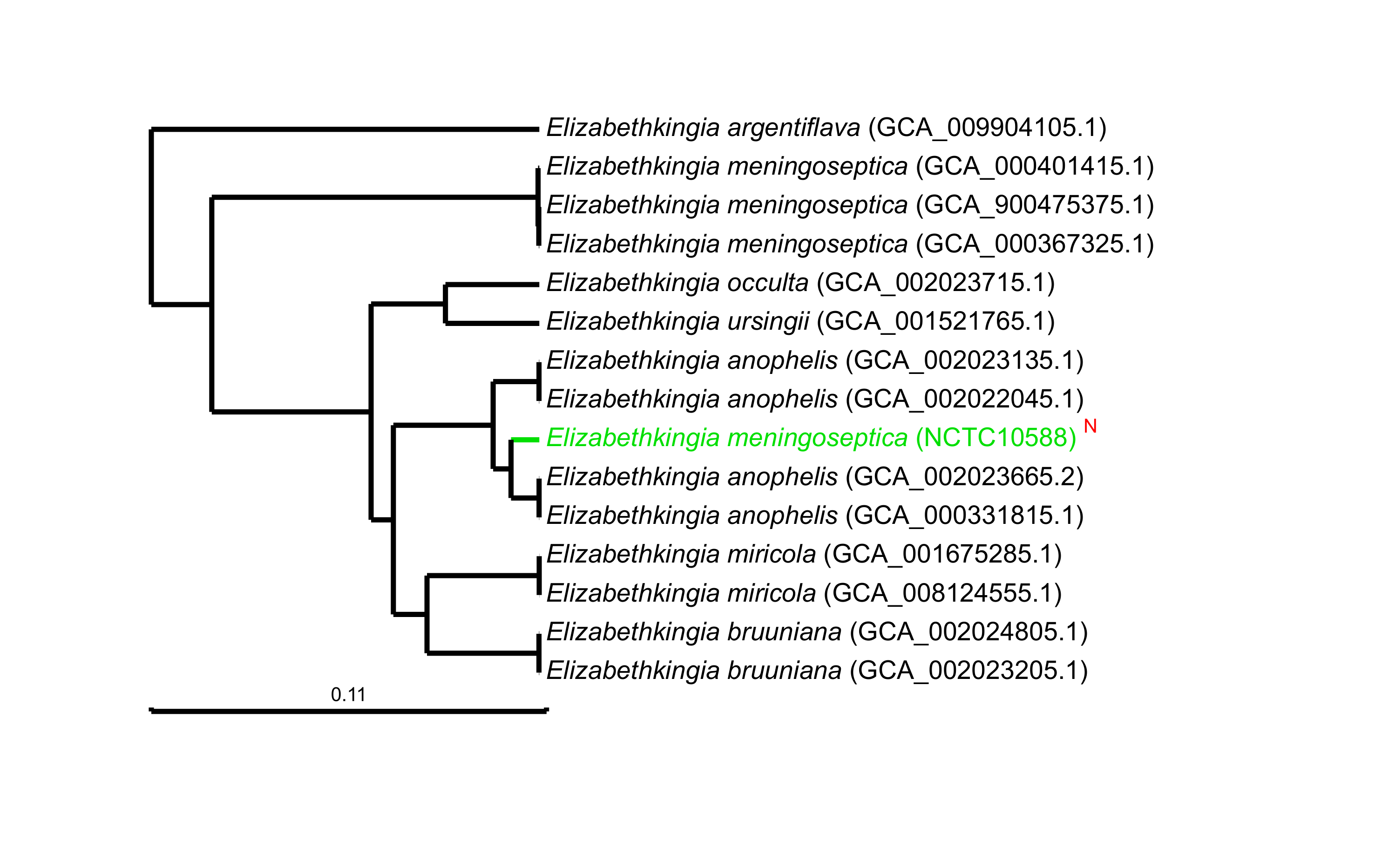

Supplement: Supplementary Figure 1 — Example phylogenetic analysis of 17 Salmonella genomes from a factory. [file Data_Sheet_1.zip › Supplementary Figure 18.Elizabethkingia.tiff]

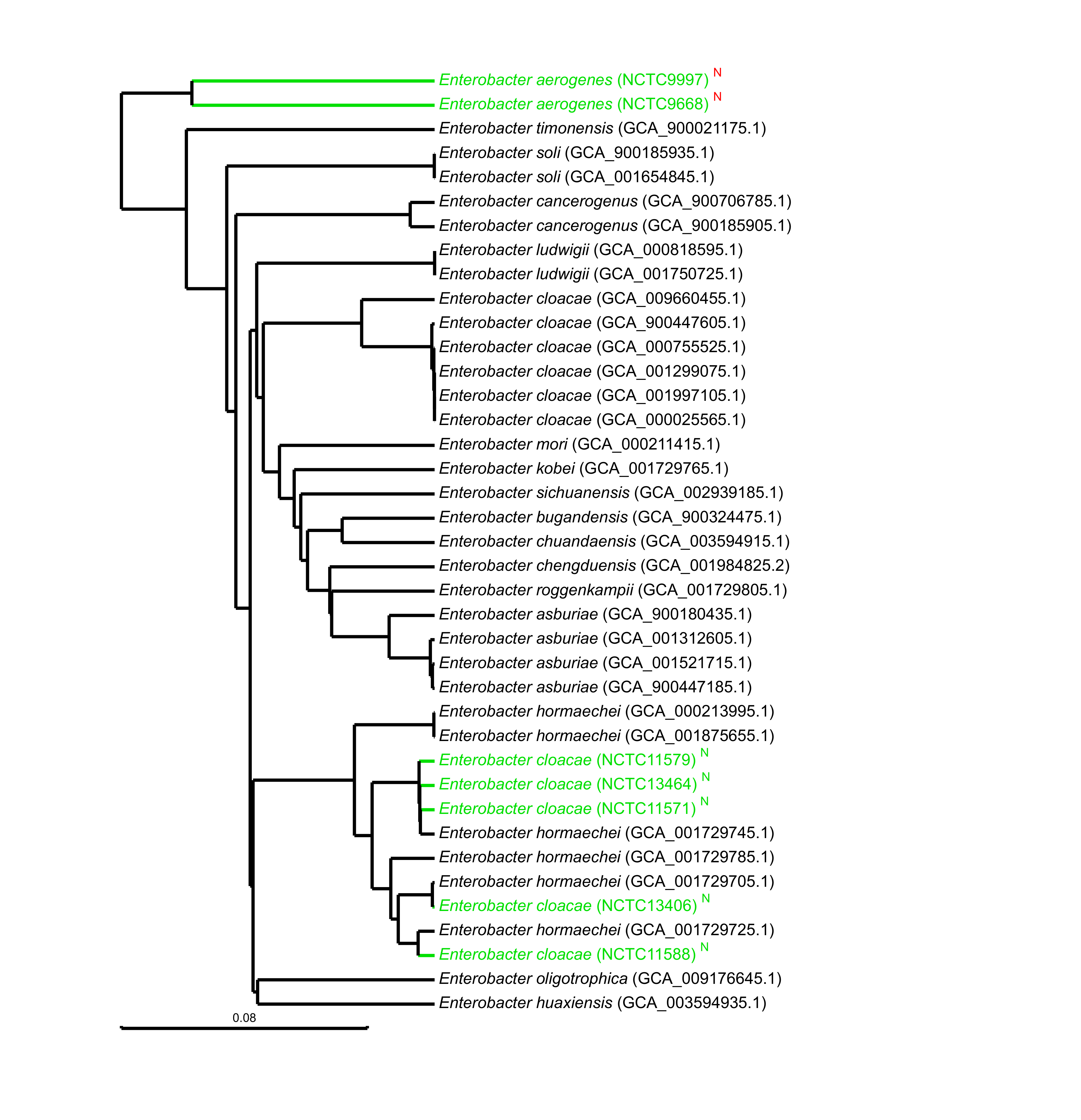

Supplement: Supplementary Figure 1 — Example phylogenetic analysis of 17 Salmonella genomes from a factory. [file Data_Sheet_1.zip › Supplementary Figure 19.Enterobacter.tiff]

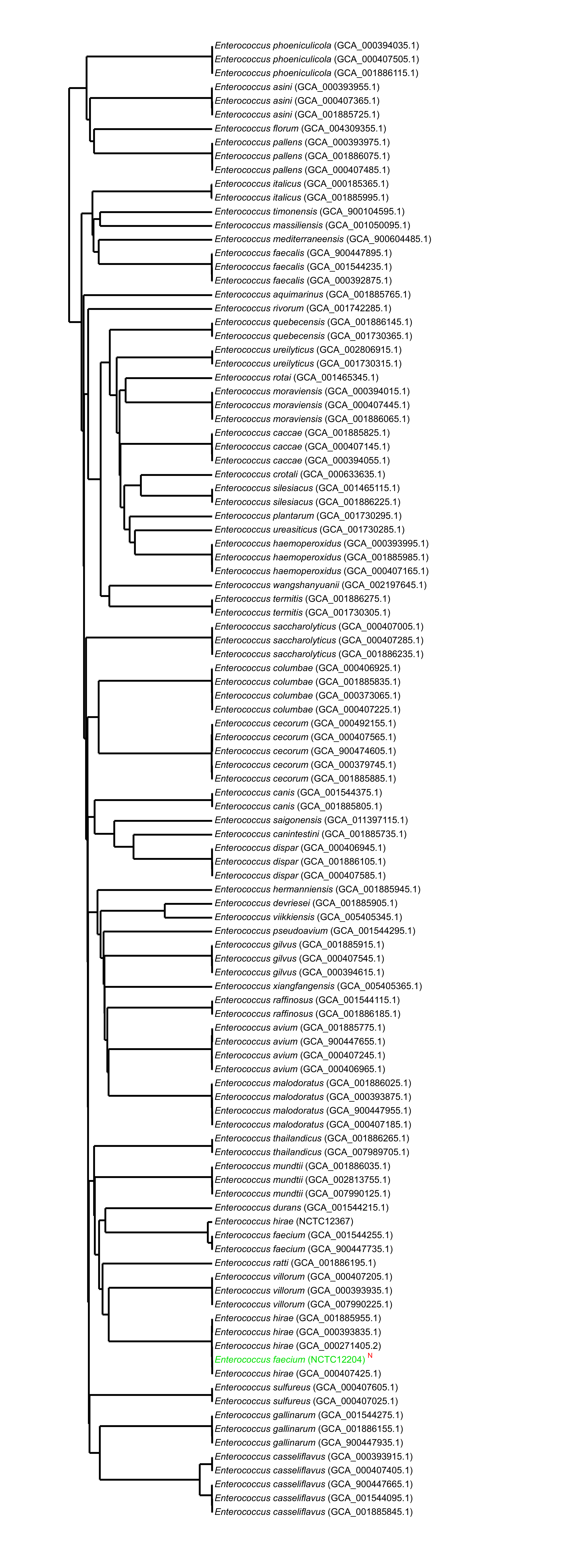

Supplement: Supplementary Figure 1 — Example phylogenetic analysis of 17 Salmonella genomes from a factory. [file Data_Sheet_1.zip › Supplementary Figure 20.Enterococcus.tiff]

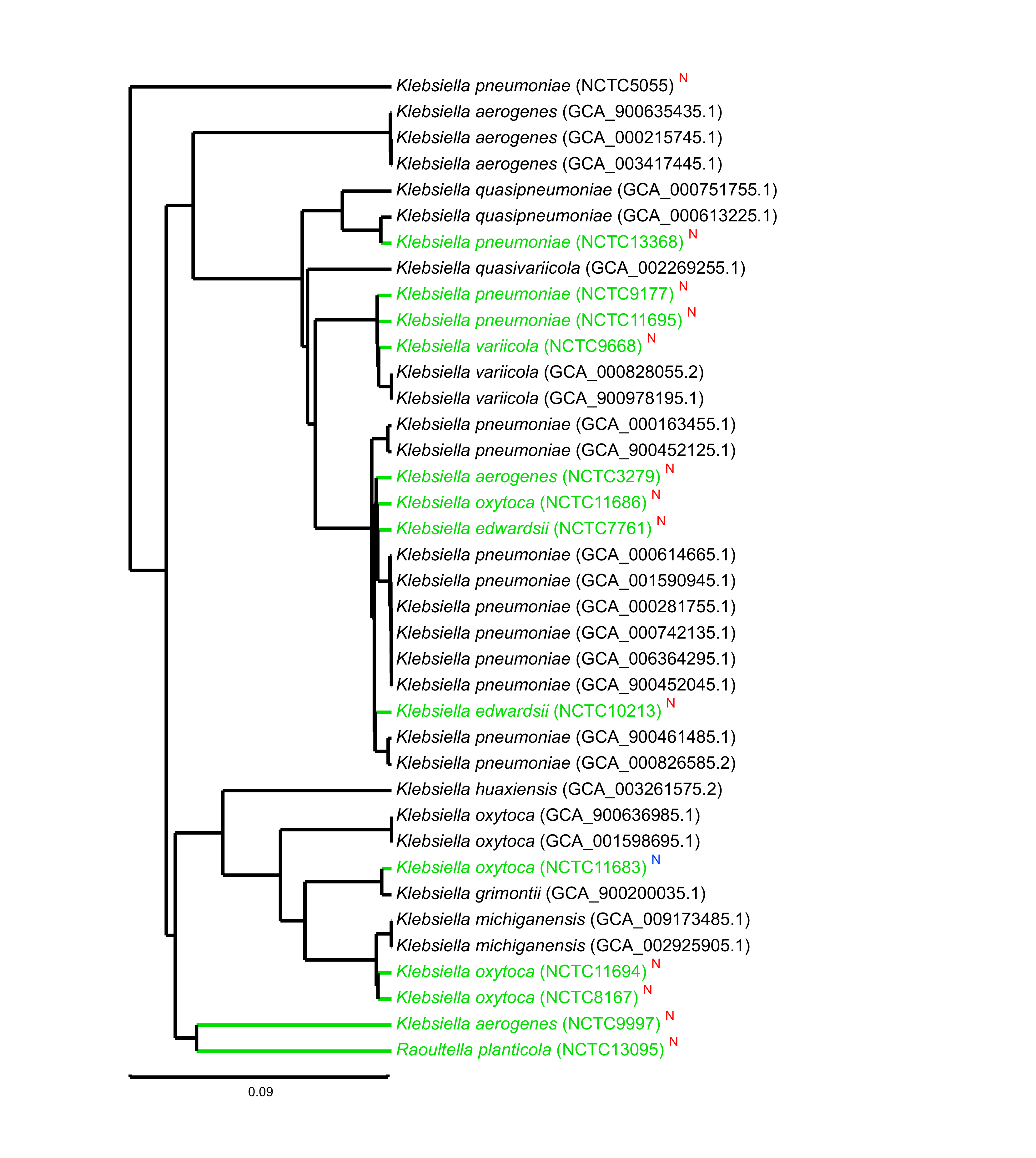

Supplement: Supplementary Figure 1 — Example phylogenetic analysis of 17 Salmonella genomes from a factory. [file Data_Sheet_1.zip › Supplementary Figure 21.Klebsiella.tiff]

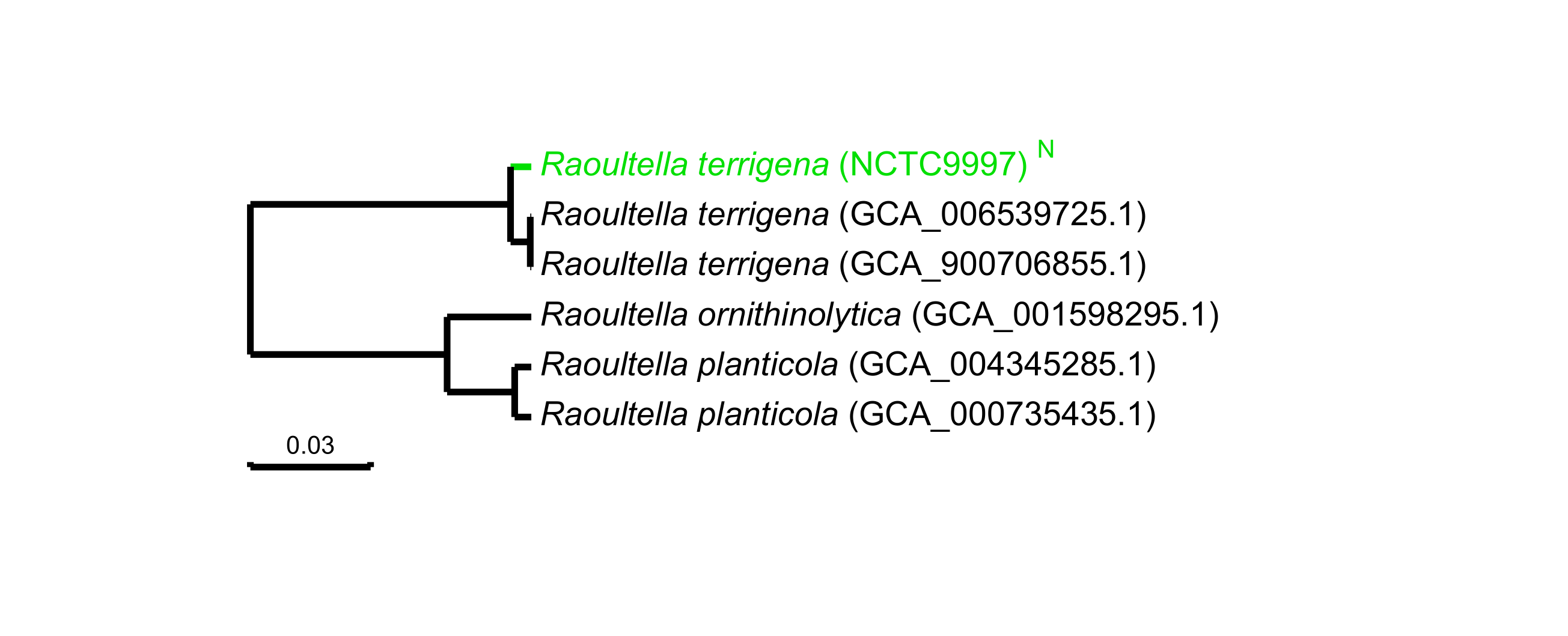

Supplement: Supplementary Figure 1 — Example phylogenetic analysis of 17 Salmonella genomes from a factory. [file Data_Sheet_1.zip › Supplementary Figure 24.Raoultella.tiff]

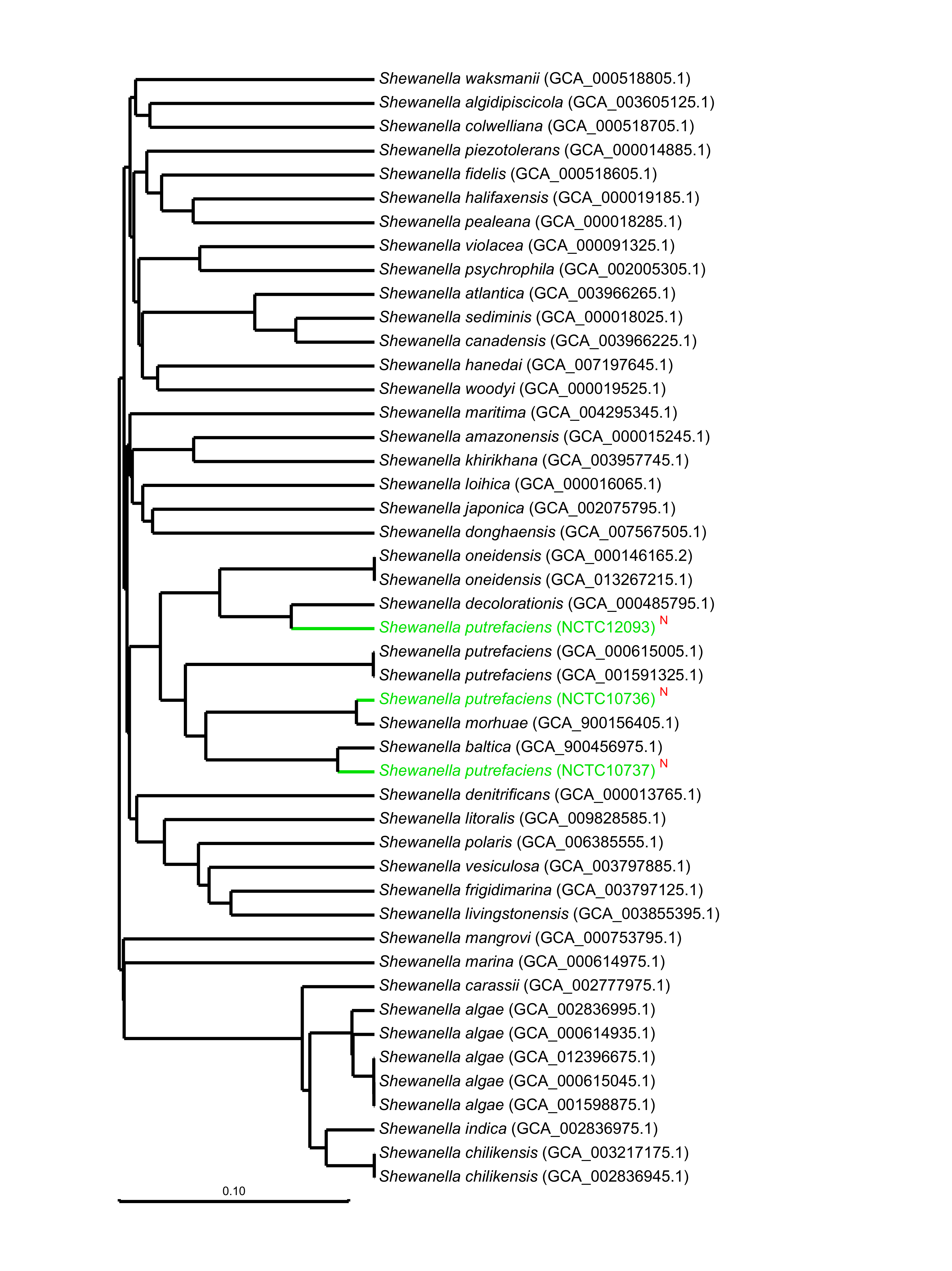

Supplement: Supplementary Figure 1 — Example phylogenetic analysis of 17 Salmonella genomes from a factory. [file Data_Sheet_1.zip › Supplementary Figure 25.Shewanella.tiff]

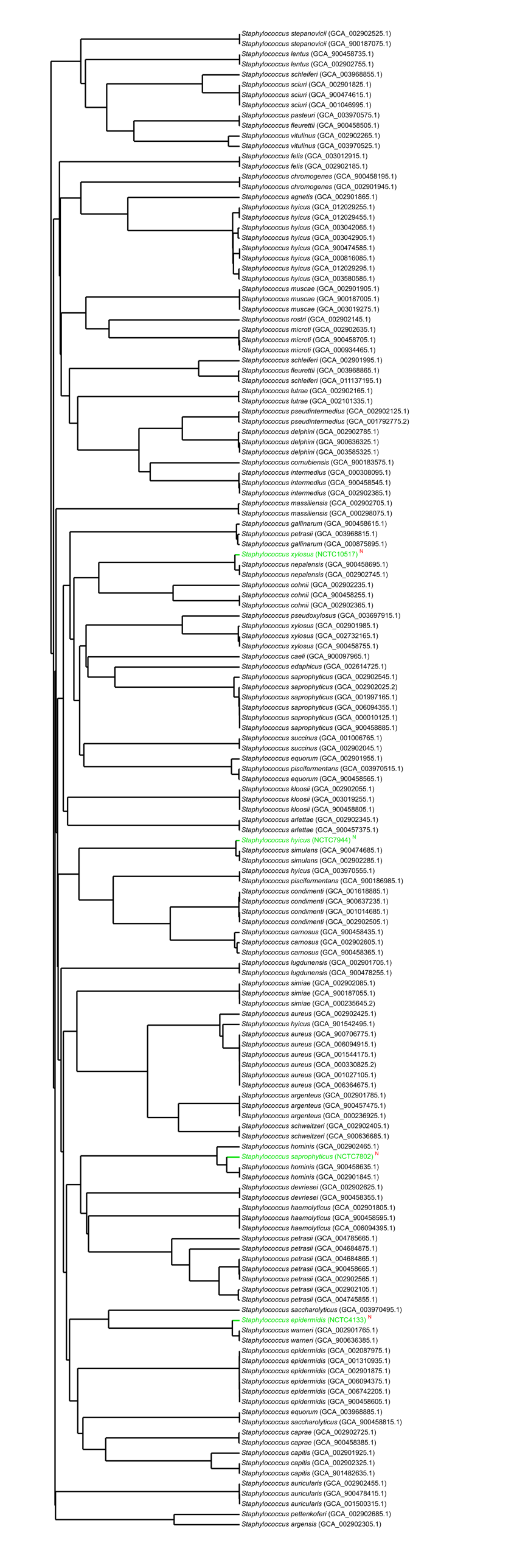

Supplement: Supplementary Figure 1 — Example phylogenetic analysis of 17 Salmonella genomes from a factory. [file Data_Sheet_1.zip › Supplementary Figure 26.Staphylococcus.tiff]

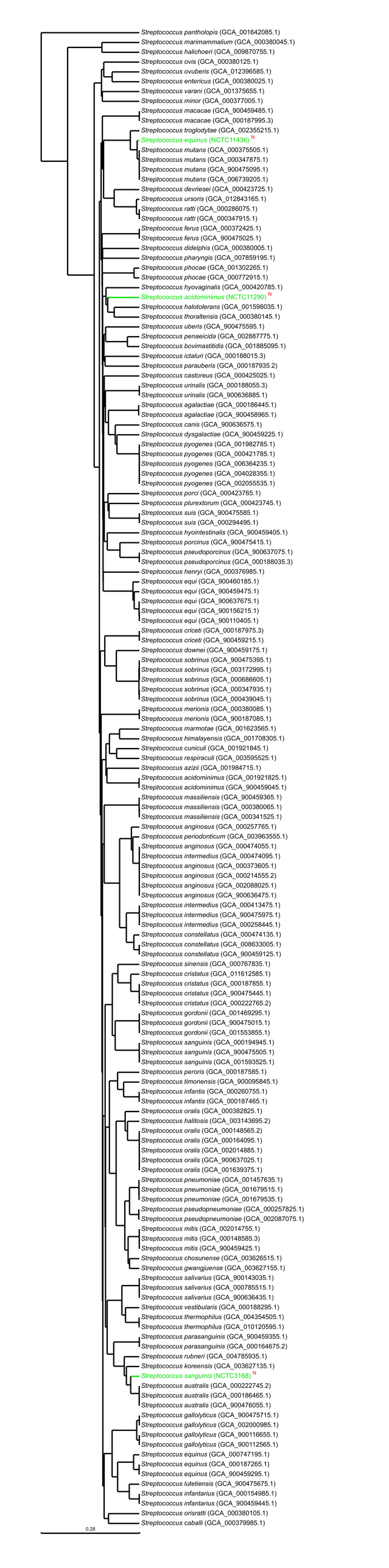

Supplement: Supplementary Figure 1 — Example phylogenetic analysis of 17 Salmonella genomes from a factory. [file Data_Sheet_1.zip › Supplementary Figure 27.Streptococcus.tiff]

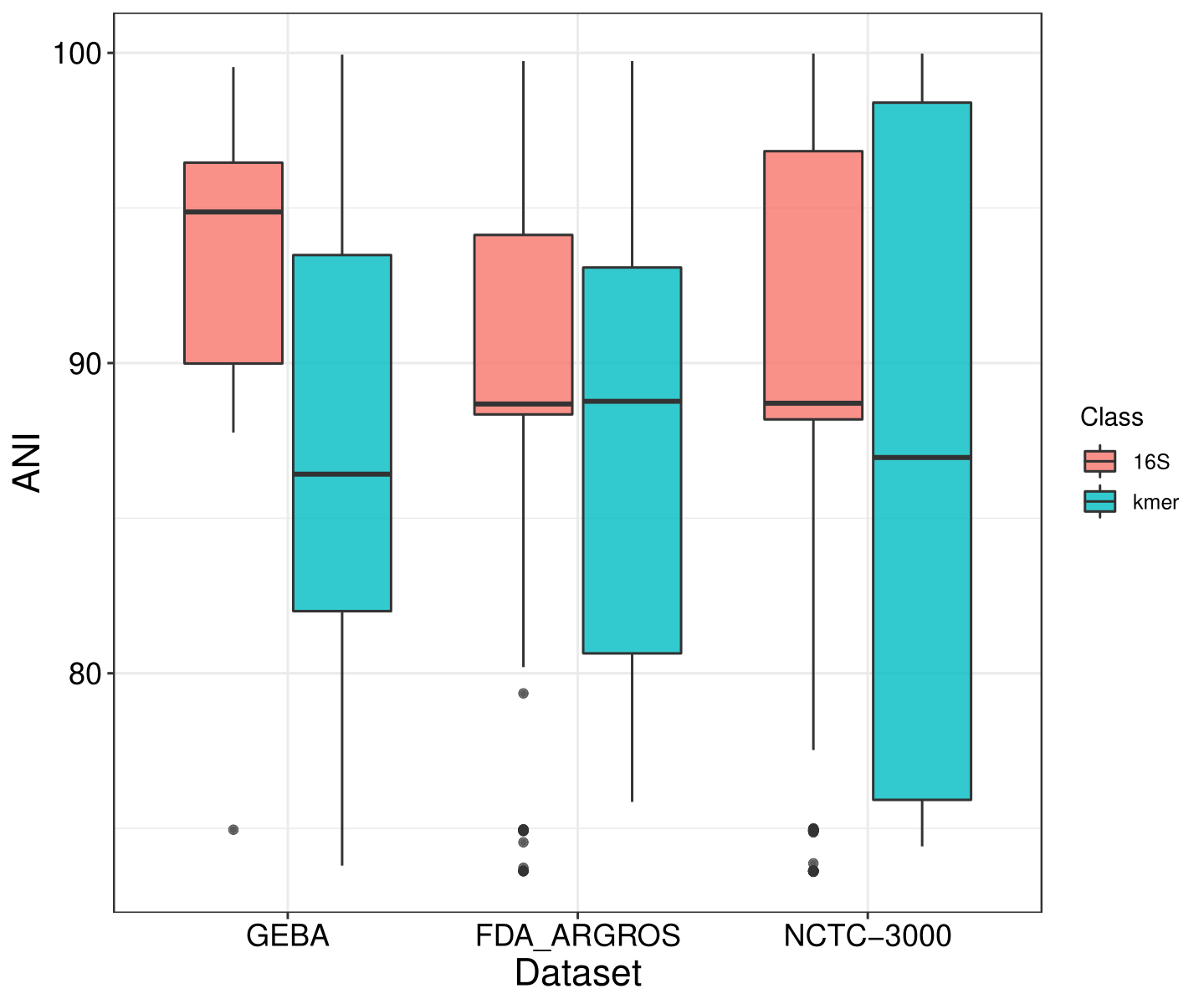

Supplement: Supplementary Figure 1 — Example phylogenetic analysis of 17 Salmonella genomes from a factory. [file Data_Sheet_1.zip › Supplementary Figure 2.tiff]

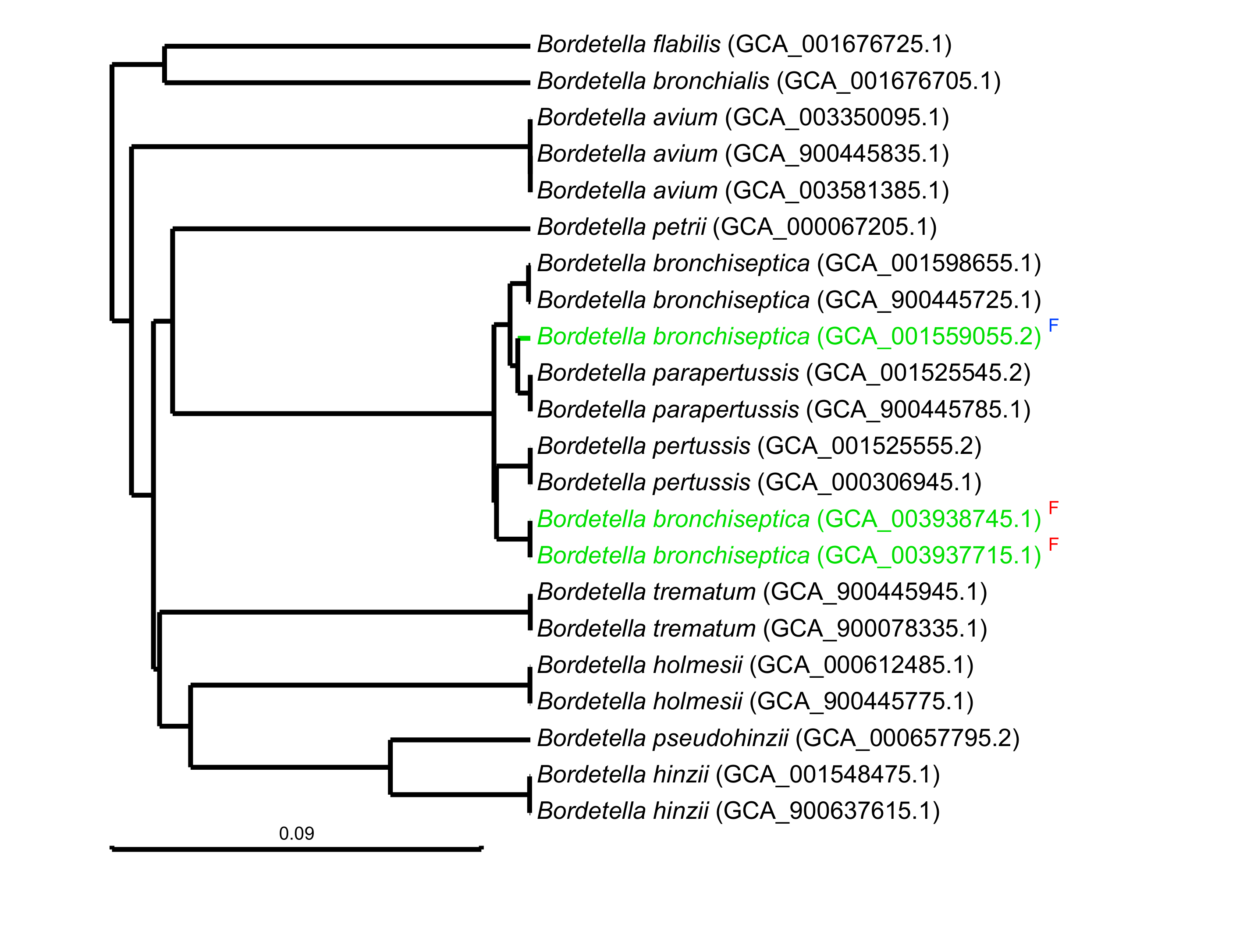

Supplement: Supplementary Figure 1 — Example phylogenetic analysis of 17 Salmonella genomes from a factory. [file Data_Sheet_1.zip › Supplementary Figure 3.Bordetella.tiff]

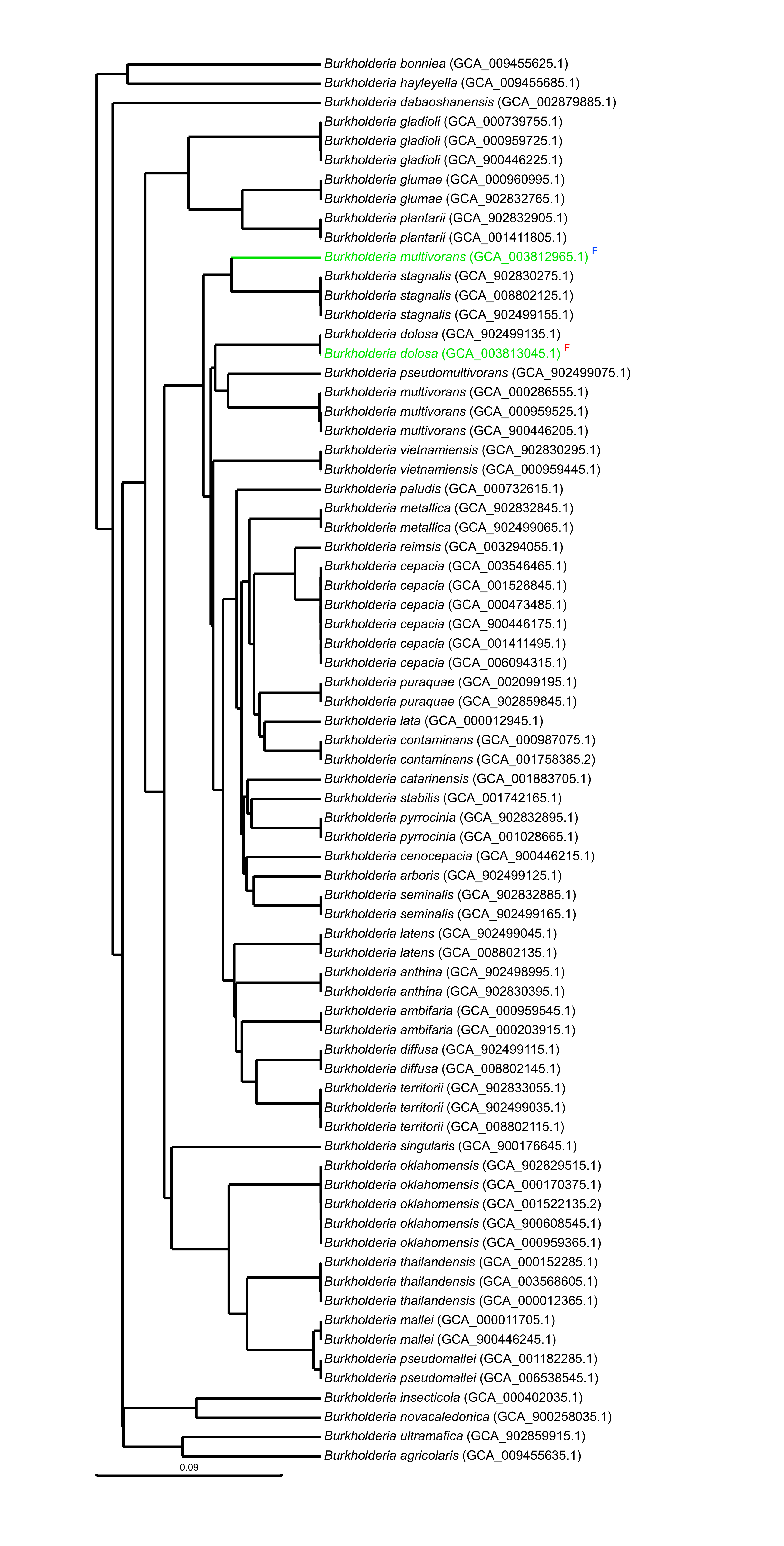

Supplement: Supplementary Figure 1 — Example phylogenetic analysis of 17 Salmonella genomes from a factory. [file Data_Sheet_1.zip › Supplementary Figure 4.Burkholderia.tiff]

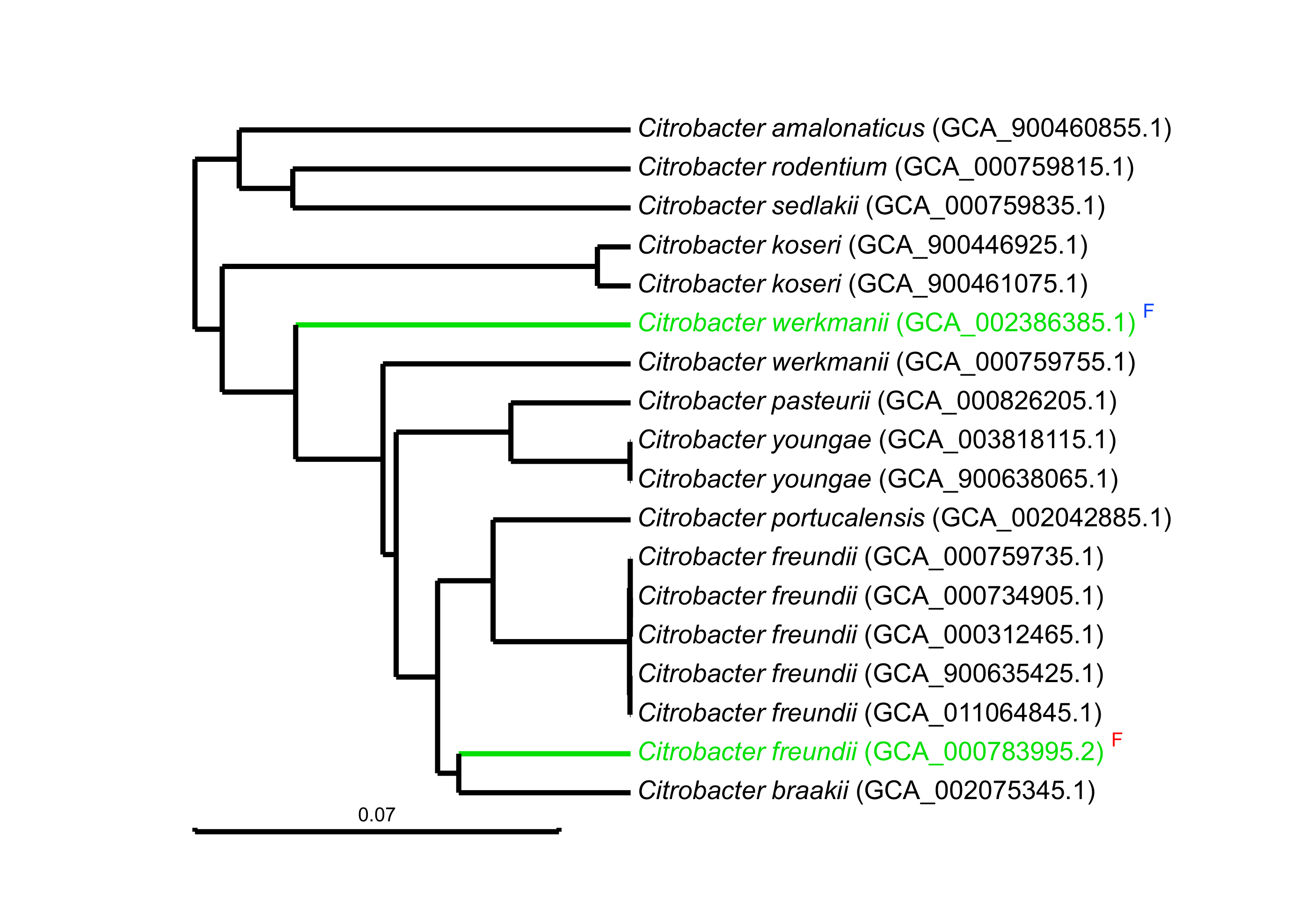

Supplement: Supplementary Figure 1 — Example phylogenetic analysis of 17 Salmonella genomes from a factory. [file Data_Sheet_1.zip › Supplementary Figure 5.Citrobacter.tiff]

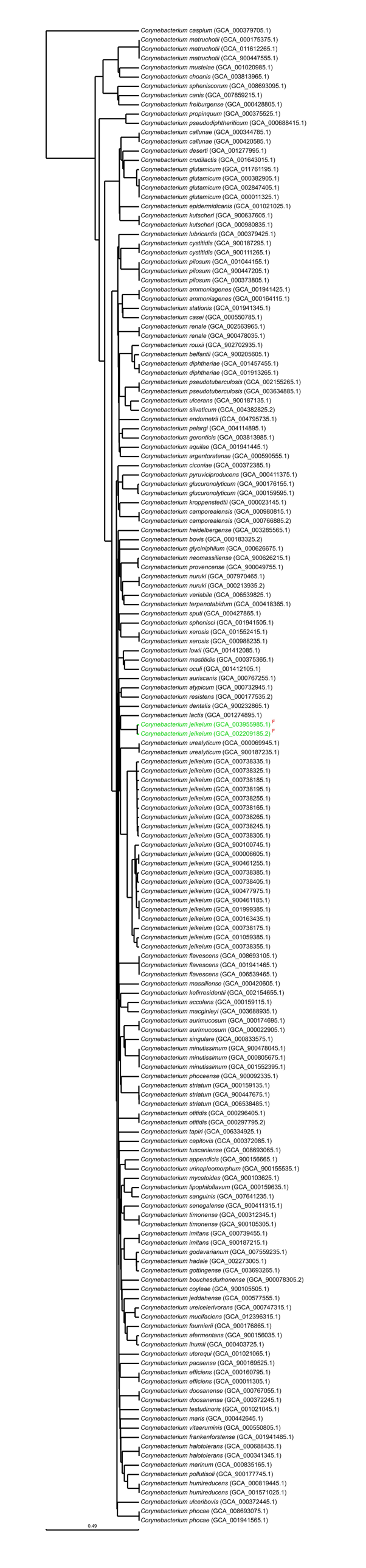

Supplement: Supplementary Figure 1 — Example phylogenetic analysis of 17 Salmonella genomes from a factory. [file Data_Sheet_1.zip › Supplementary Figure 6.Corynebacterium.jpg]

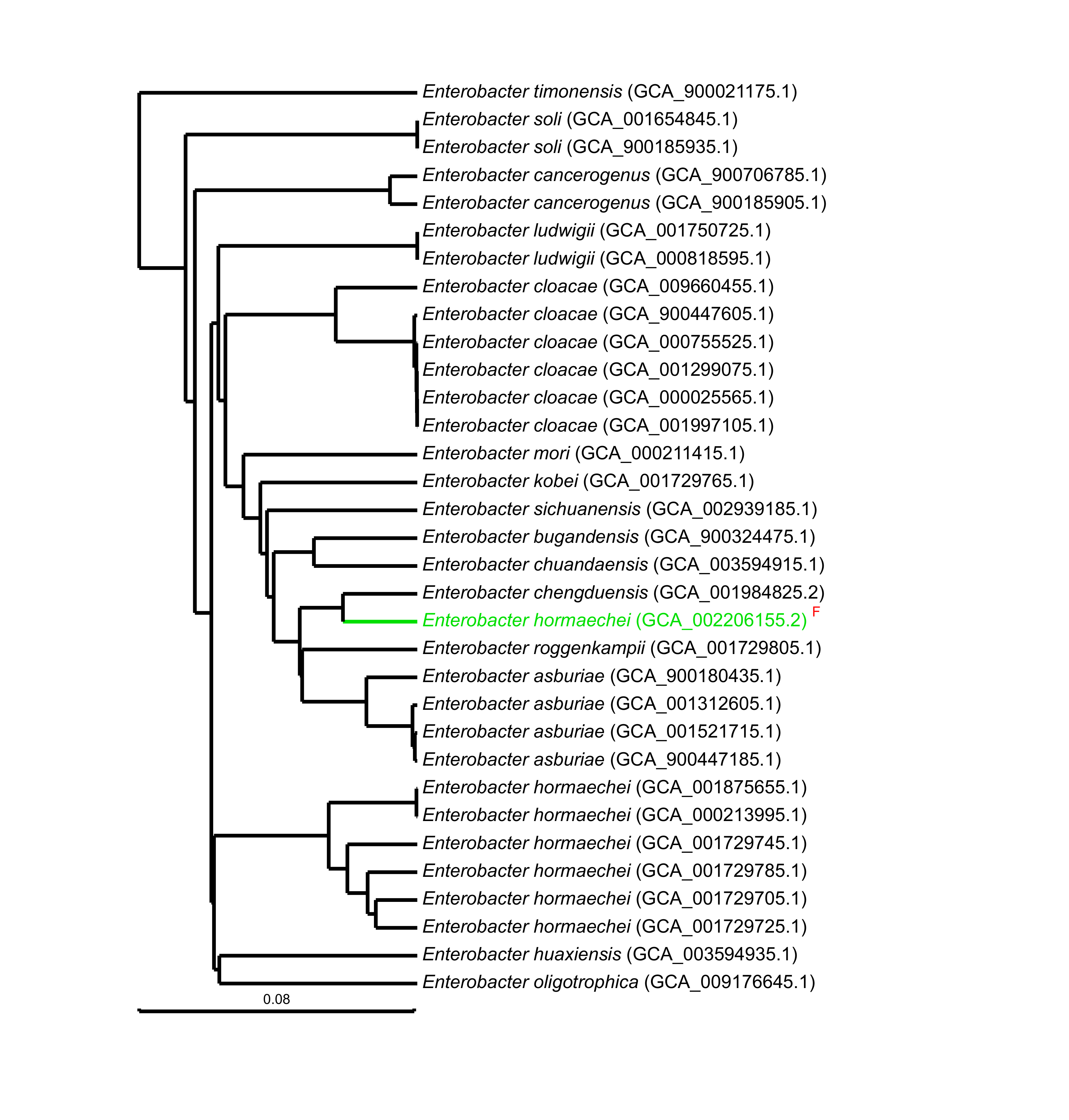

Supplement: Supplementary Figure 1 — Example phylogenetic analysis of 17 Salmonella genomes from a factory. [file Data_Sheet_1.zip › Supplementary Figure 7.Enterobacter.tiff]
